# An adaptive dynamics framework for microbial ecology and evolution

**DOI:** 10.1038/s41598-025-08636-5

**Published:** 2025-07-07

**Authors:** Carl-Joar Karlsson, Philip Gerlee, Julie Rowlett

**Affiliations:** Department of Mathematical Sciences, Chalmers University of Technology and the University of Gothenburg, 412 96 Gothenburg, Sweden

**Keywords:** Adaptive dynamics, Nash equilibrium, Evolutionary game theory, Non-cooperative game theory, Dynamical systems, Microbe ecology, Plankton, Pure mathematics, Applied mathematics, Microbial ecology

## Abstract

Adaptive dynamics describes a deterministic approximation of the evolution of scalar- and function-valued traits. We construct an evolutionary process for a game-theoretic model which may describe the evolution of microbes. In our analysis, we demonstrate the existence of solutions to the adaptive dynamics and determined their regularity. Moreover, we identify all stationary solutions and prove that these are precisely the Nash equilibria of the game theoretic model. Numerical examples are provided to highlight the main characteristics of the dynamics. The dynamics are unstable; non-stationary solutions oscillate and perturbations of the stationary solutions do not shrink. Instead, a linear type of branching may occur. This may explain the ever-increasing complexity in microbial biological systems and provide a mechanistic explanation for not only the tremendous biodiversity observed in microbe species but also for the extensive phenotypic variability within species.

## Introduction

Biological diversity is a striking feature of many ecosystems and is known to provide stability and resilience^1^. It can be affected by external factors such as the resource availability^2^, and the amount of green area in cities^1^, but it is also a consequence of the evolutionary process itself. There is however a need to better understand the intrinsic processes that lead to biological diversity in the absence of external influence. Mathematical modeling and the simplifying assumptions that accompanies it has sometimes led to unrealistic conclusions, for example that the number of species cannot exceed the number of limiting resources^3^. Similarly, the so called exclusion principle says that two competing species that occupy the same ecological niche cannot co-exist^4^.

For marine microbes, the number of microbial species by far exceeds the predictions from competition theory, and there is tremendous variability between and within species^5–10^. This discrepancy between theory and reality is known as the “paradox of the plankton” in marine ecology and it has gained considerable attention^11^. Attempts to resolve the paradox abound. For instance, Huisman and Weissing^12^ demonstrated that common competition models can sustain a system with large numbers of species by oscillating, or cycling, the population sizes. This would partly solve the paradox of the plankton, since the oscillating populations can coexist in much higher numbers than predicted by steady-state analysis. Other mechanisms that have been suggested include spatio-temporal heterogeneity^13^, viral lysis^14^ and size-selective grazing^15^.

A different approach was taken by Menden-Deuer and Rowlett^7,16–18^ when they modeled the inter-species competitions among asexually reproducing microbes using non-cooperative game theory. The game accounts for intra-species heterogeneity and each individual is equipped with a competetive ability (CA), which is drawn from a species-level distribution. Individuals are paired-off randomly against each other and the expected species-level pay-off can be computed. In the case where the mean competitive ability is bounded it can be shown that an unlimited number of species may coexist.

However, the game-theoretic resolution put forth by Menden-Deuer and Rowlett to the paradox of the plankton only offers an equilibrium perspective of the problem. It explains *that* many competing species can coexist, but says nothing about *how* diversity appeared in the first place or how it evolves over time. Or in other words, we still lack an understanding of the evolutionary dynamics of species that interact according to the above mentioned game.

A dynamic description of evolution is offered by the framework of adaptive dynamics^19,20^, which describes how a certain trait in a population changes due to the invasion of successive mutants. In order to make use of the theory of adaptive dynamics the following assumptions are required: i) mutations are rare enough so that competition is always between two subpopulations – the resident and a mutant, ii) the initial growth rate of the mutant when rare (also called its invasion fitness) is sufficient to determine the outcome of the competition between the resident and the mutant and iii) mutations to the considered trait are small and hence the gradient of the invasion fitness provides a description of the dynamics.

Adaptive dynamics has successfully been applied to cases where the trait is described by a single value, e.g. body weight. Equilibrium points in trait space where the fitness gradient vanishes can be characterised according to if they are stable to perturbations (evolutionary stability) and if they are reachable by the dynamics (convergence stablility). Points that are convergence stable, but not evolutionary stable allow for evolutionary branching, which can be viewed as a precursor of speciation.

Many traits cannot be captured by a single value, and also seemingly different traits might have a common genetic basis implying that mutations cause them to co-vary. In those cases one needs to resort to vector-valued adaptive dynamics, which describes evolution in a multi-dimensional trait space^21^. The above mentioned characterisation of equilibrium points has been extended to the multi-dimensional case^22^.

However, sometimes not even a vector-valued description is sufficient when the trait varies smoothly, e.g. the response to environmental conditions such as salinity. In such cases, a function-valued version of adaptive dynamics is required^23,24^. Here we will make use of both vector-valued and function-valued adaptive dynamics for studying the evolution of the game introduced in^7,16–18^. In addition we formulate the function-valued dynamics as a gradient flow. We rigorously prove mapping properties of the selection gradient acting on function spaces, and we prove existence and regularity results for the dynamical system. We suggest that our analysis of the vector-valued and function-valued adaptive dynamical systems, which are related but nonetheless present distinct features, demonstrate how one may rigorously analyze adaptive dynamical systems in other specific contexts.

The manuscript is organised as follows. The Results section contains the definitions of the games and the main results for the adaptive dynamical systems. It is followed by the Discussion, which puts the results into context. The Methods section contains proofs and more technical lemmas and propositions.

## Results

To explain our results we must first explain the games for which we create an adaptive dynamical system. The game introduced by Menden-Deuer and Rowlett^7,16,17^ was initially developed to investigate asexually reproducing microbe species competing for survival. Later, it was generalized and interpreted in other contexts^18^. To describe this game, we consider a collection of species, each consisting of several individuals. Each individual has a “strength” that can be measured and compared with the strength of another individual. This strength is known as *competitive ability*^17^, abbreviated CA. When species compete, their cumulative wins and losses are assessed amongst all individuals of each species in order to determine the payoff to each species. Initially, competitive abilities were selected from a discrete set of values, allowing the game to be expressed using vectors to represent strategies. We first introduce this vector-valued game.

### Introducing the vector-valued game

In this game the competitive abilities are selected from the values $\{k/M\}$ for the integers $k=0, \ldots , M$. For simplicity, assume that there are just two species. The species compete in such a way that one randomly chosen individual from one species competes against an individual from the other species, which is also chosen at random. The stronger individual defeats the weaker so that it can replicate, while the losing individual dies. Nothing happens if the competitors are equally strong. Thus, individual success implies population growth of the species to which the winning individual belongs, while the losing species experiences a population decrease. This individual competition repeats, and the cumulative losses and gains can result in either one’s extinction and the other’s dominance or co-existence.

Let $y_k$ be the number of individuals in species $\varvec{y}$ with competitive ability equal to $\frac{k}{M}$, and $z_k$ be the number of individuals in species $\varvec{z}$ with competitive ability equal to $\frac{k}{M}$. For this to be meaningful we assume

2.1 $$\begin{aligned} y_k, z_k \in [0, \infty ), \quad \forall 0 \le k \le M, \quad \sum _{k=0} ^M y_k> 0, \quad \sum _{k=0} ^M z_k> 0. \end{aligned}$$

Note that $y_k$ and $z_k$ do not need to be integer-valued. Then, the payoff in the game as described above to $\varvec{y}$ in competition with $\varvec{z}$ is

2.2 $$\begin{aligned} E[\varvec{y}, \varvec{z}] = \sum _{k=0}^M y_k \left( \sum _{j=0}^{k-1} z_j - \sum _{\ell =k+1}^M z_\ell \right) . \end{aligned}$$

The payoff to $\varvec{z}$ in competition with $\varvec{y}$ is computed analogously, by summing over the cumulative wins and losses, so that

$$\begin{aligned} E[\varvec{z},\varvec{y}] = \sum _{k=0}^M z_k \left( \sum _{j=0}^{k-1} y_j - \sum _{\ell =k+1}^M y_\ell \right) . \end{aligned}$$

It is straightforward to compute that this game is zero-sum and symmetric. Each competition between two species has randomly selected individuals competing, but $E[\varvec{y},\varvec{z}]$ captures the statistical success of the competing species and can be analyzed without addressing the randomness of the game. Identifying each CA value as a pure strategy, the vector $\varvec{y}=(y_0, \ldots , y_M)$, suitably normalized, can be identified with a mixed strategy, and the game can be expressed in normal form such that $E[\varvec{y},\varvec{z}]$ is the payoff to $\varvec{y}$ in competition with $\varvec{z}$ computed according to the definition of expected value. The strategy of a species assigns competitive abilities to individuals in the sense that

$$\begin{aligned} \frac{y_k}{\sum _{k=0} ^M y_k}\end{aligned}$$

is the probability that a randomly selected individual from species $\varvec{y}$ has competitive ability equal to $\frac{k}{M}$. One could instead define $y_k$ to be equal to this probability, which simply amounts to dividing $y_k$ as defined here by the total number of individuals; see also the explanation on^16^, p. 5. The results obtained here would not change, but without this normalization the mathematical proofs are clearer, so for that reason we do not make this normalization.

For the game to be fair and interesting, we impose that every species must respect a bound on its mean strength, or *mean competitive ability, *abbreviated MCA. This MCA for species $\varvec{y}$, as well as its constraint are respectively

2.3 $$\begin{aligned} {{\,\textrm{MCA}\,}}(\varvec{y}) := \frac{\sum _{k=0}^M \frac{k}{M} y_k}{\sum _{k=0}^M y_k} \le \frac{1}{2}. \end{aligned}$$

The same constraint is imposed on any other species that competes. We will identify a species with its strategy, since the strategy of the species fully characterizes and distinguishes the species. The strategy can be uniquely identified with a vector in $\mathbb {R}^{M+1}$ whose components satisfy (2.1) and (2.3). One way to create a species is to compute the sum of two species, which means that we compute the sum of their strategies because the resulting strategy will satisfy both (2.1) and (2.3). In this way, one may also consider any number of competing species by letting each species compete against the sum of all the others. The only restrictions on the composition of species are (2.1) and (2.3); otherwise, they can be freely chosen.

The choice of upper bound in (2.3) is more general than one might think, as we have observed in our prior work^18^. In the current game, one could equally well work with competitive abilities contained in any bounded interval, or any interval bounded from below, and with any constraint value. It is proven in^18^ that this leaves the main results concerning the Nash equilibria as well as the characterization of strategies in the game in terms of their wins and/or losses unchanged. Consequently, this choice similarly does not affect the associated dynamical system. We will therefore concentrate on the setting which fixes the upper bound on the MCA at 1/2, that is, as in equation (2.3), and with competitve abilities contained within the interval [0, 1].

To identify those strategies that may be more likely to win in competition with others (or less likely to lose), we recall an important notion in game theory, an *equilibrium point, *also known as a *Nash equilibrium point *due to Nash’s proof of their existence^25^. An equilibrium point is a collection of strategies for all competing species, so that if any one species alone changes their strategy, their payoff does not increase. Menden-Deuer *et al.*^16^ identified all equilibrium points for this game. We summarize the result here.

#### Theorem 2.1

In the vector-valued game as defined here, assume first that *M* is odd. Then an equilibrium point consists of strategies that are a positive scalar multiple of the vector $(1, 1, \ldots , 1)$. If we instead assume that *M* is even, then an equilibrium point consists of strategies that are of the form $(a,b,a,b, \ldots , a)$ for two non-negative constants *a* and *b* that are not both zero.

The phenomenon that the shape of equilibrium strategies depends on the discretization of the game motivates us to consider a game in which the values of competitive ability can be selected from the entire (continuous) range of values [0, 1]. This leads us to the function-valued game.

### Introducing the function-valued game

Completely analogous to the discrete strategies are the *continuous* and *bounded measurable* strategies, introduced by Menden-Deuer *et al.*^16^. For a continuous (respectively, bounded measurable) nonnegative function defined on [0, 1], we use the measure *f*(*x*)*dx* with *dx* the one-dimensional Lebesgue measure to define the quantity of individuals of the associated species having competitive ability within any given subinterval of [0, 1]. Analogously to the vector-valued game, we identify a species with its strategy, which is a function satisfying

2.4 $$\begin{aligned} f:[0,1] \rightarrow [0, \infty ), \quad \int _0 ^1 f(x) dx> 0, \quad {{\,\textrm{MCA}\,}}(f) = \frac{\int _0 ^1 x f(x) dx}{\int _0 ^1 f(x) dx} \le \frac{1}{2}. \end{aligned}$$

In the continuous game, we assume further that the function is continuous, whereas in the bounded measurable game, we only assume further that the function is in $L^\infty [0,1]$. We refer to both of these games as *function-valued games. *

The payoff to a strategy *f* in competition with a strategy *g* is in this case

$$\begin{aligned} E[f,g] = \int _0^1 f(x) \left( \int _0^x g(y)\,dy-\int _x^1 g(y)\,dy \right) dx \end{aligned}$$

The game in this case also generalizes to multiple species analogously to the vector-valued game. Specifically, *f* competes with *n* other species $g_1,...,g_n$ by competing with the strategy defined by the sum of the other species, noting that this strategy satisfies (2.4). Menden-Deuer *et al.*^16^ identified the equilibrium points for these games.

#### Theorem 2.2

In the function-valued game, all equilibrium points are collections of strategies for all teams that are positive constant functions that are not necessarily identical.

The restriction to [0, 1] in all games here can be relaxed; the functions can be supported on any compact subset of the real line. However, the unit interval is convenient and if a function is defined in any other compact interval on the real line, then it can be transformed by a change of variables to a function on [0, 1]. So, no generality is lost by making this assumption. For details, see^18^.

### Evolutionary stable strategies do not exist for these games

We recall that an evolutionary stable strategy (ESS) must be a strategy *S* such that for all strategies $T \ne S$ we have either:

1. $$E[S,S]> E[T, S]$$ or
2. $$E[S,S] = E[T,S]$$ and $$E[S, T]> E[T, T]$$.

Our first result shows that these games do not admit any evolutionary stable strategies. The proof of this result is based on Theorems 2.2 and 2.1.

#### Corollary 2.3

In both the function-valued and vector-valued games, there are no evolutionary stable strategies.

#### Proof

These games are zero-sum and symmetric, and so the first condition to be an ESS would require the existence of a strategy *S* such that for any strategy $T \ne S$ we have

$$\begin{aligned} 0> E[T,S] = - E[S, T] \implies E[S, T]> 0. \end{aligned}$$

In the proofs of Theorems 2.2 and 2.1 we showed that there is no such strategy. The second statement in the definition of ESS would require

$$\begin{aligned} 0 = E[S, S] = E[T, S] = - E[S, T] \implies E[S, T] = E[T, T] = 0. \end{aligned}$$

Thus $E[S, T]> E[T, T]$ is impossible. $\square$

This result indicates the the team game evolves endlessly under the adaptive dynamics framework. As will be shown later, the precise mathematical statements are: There are dynamical equilibria consisting of constant functions, but these are not stable. A small perturbation of an equilibrium will unsettle the system into evolution. The adaptive dynamics does not incorporate such perturbations automatically, but knowing that equilibria are not stable and by interpreting the deterministic dynamics as approximations of a natural system which contains demographic noise, it is reasonable to argue that the lack of ESS and dynamically stable equilibria must lead to continuous evolution. These biological interpretations appear to be consistent with empirical observations^7,16,17^.

### Adaptive dynamics

At the heart of adaptive dynamics lies the assumption that there exists a “resident population” in which mutations can appear and that the success of mutants can be inferred from the initial growth rate of the mutated individuals. The initial growth rate of mutants is called *invasion fitness*. In addition, mutations are assumed to be rare, so that each mutant either takes over the entire population or goes extinct before the next mutant arrives^20,26,27^. In other words, if a mutant has lower fitness than the resident population, then it disappears, but if the mutant’s fitness is higher than the resident population’s, then it is assumed that the mutation spreads into the entire resident population. By assuming this, adaptive dynamics offers a deterministic description of biological evolution.

Dieckmann *et al.*^24^ proposed a framework for adaptive dynamics on function-valued traits. They used approximations to stochastic models, assuming that (a) mutations make small changes to the traits and that (b) the natural selection occurs much faster than the typical time between the appearance of novel mutations, so that each population is monomorphic. The result of these considerations is that a trait function *f* develops according to

2.5 $$\begin{aligned} \frac{d}{dt} f(x) = \frac{1}{2}\mu _f \bar{n}_f \int _\Omega \sigma ^2_f(x,y) g_f(y)\,dy \end{aligned}$$

where *f* is the trait/strategy. The integration domain $\Omega$ must be selected to suit the model. The quantity $\mu _f$ is the probability that *f* can be reached by mutations of nearby strategies, and $\bar{n}_f$ is the equilibrium population size, which is assumed to be constant and independent of the strategy *f*. Here, $\sigma ^2_f$ is the variance-covariance function of the mutation distribution. The role of the variance-covariance function $\sigma ^2_f$ is to account for cross-dependence; if the dynamics at *x* changes the strategy *f* in such a way that it affects *f* at another point *y*, this is encoded in the variance-covariance function. Typically, this is formulated as a constraint on all traits. The function $g_f$ is the functional gradient of the invasion fitness function. Let *S* be the set of strategies and let *E*(*f*, *g*) be the invasion fitness of $f\in S$ in the resident population with trait $g\in S$. Then

$$\begin{aligned} g_f(x) = \left. \frac{d}{dt}\right| _{t=0} \, E(f+t\delta _x,f). \end{aligned}$$

Here, $\delta _x$ is the Dirac delta distribution^28^ centered at *x*. We will call the dynamics equation (2.5) the *canonical equation* of adaptive dynamics of function-valued traits. Although we first introduced the vector-valued game, adaptive dynamics applies more readily to the function-valued game. For this reason we continue with the derivation of the adaptive dynamical system for the function-valued game. Moreover, this derivation will be instructive in showing how to define the dynamical system for the vector-valued game.

### Adaptive dynamics of the function-valued game

Consider a collection of several species: $f_1, \ldots , f_n$, each of which is characterized by its strategy $f_i$. A situation in which they all compete in the same game can be interpreted as a competition between species (or strains of a species) for resources or in combat or similar situations. Thus, it is in complete analogy with Geritz *et al.*^27^ and Dieckmann *et al.*^24^ that we let $E[f_i,f_1+f_2+...+f_n]$ be the growth rate of a species (or strain) *i* in competition with all other species. Notice that

$$\begin{aligned} E[f_i,f_1+f_2+...+f_n]&= E[f_i,f_1]+E[f_i,f_2]+...+E[f_i,f_n]\\&= E[f_i,f_1]+E[f_i,f_2]+...+E[f_i,f_{i-1}]+E[f_i,f_{i+1}]...+E[f_i,f_n] \end{aligned}$$

since $E[f_i,f_i]=0$. Moreover, if *a* is a constant, and *f*, *g* are two integrable functions, then $E[a f,g]=a E[f,g]$ and $E[f,a g]= a E[f,g]$. The selection gradient is therefore given by

2.6 $$\begin{aligned} g_f(x)=\left. \frac{d}{dt}\right| _{t=0} E[f+t\delta _x,f] = \int _0^x f(y)\,dy - \int _x^1 f(y)\,dy. \end{aligned}$$

The canonical equation of adaptive dynamics, equation (2.5), now reads

2.7 $$\begin{aligned} \frac{d}{dt} f(x) = \frac{1}{2}\bar{n}_f\mu _f\int _0^1 \sigma ^2_f(x,y)\left( \int _0^y f(z)\,dz - \int _y^1 f(z)\,dz\right) dy. \end{aligned}$$

We will assume that $\bar{n}_f$ is a constant, since it appears as a prefactor in equation (2.5) and therefore only impacts the *rate* of change and not the *direction*. We assume that competitive ability does not affect mutation rate and therefore mutation probability $\mu _f$ in the canonical equation (2.5) will also be assumed to be constant. As there are no physical time units in the canonical equation, we may set $\frac{1}{2}\bar{n}_f\mu _f=1$ without losing any information. Notice that in the canonical equation (2.5), we have $\Omega =[0,1]$.

In this case, and if it is further assumed that there is no variance or covariance, the function-valued strategies would develop in time according to the gradient flow equation

$$\begin{aligned} \frac{d}{dt}f = g_f(x). \end{aligned}$$

This clearly does not take into account that *f* may be subject to model-specific constraints. In computing the selection gradient, see equation (2.6), we use the Dirac delta, which is a distribution. However, it is also possible to derive the expression for the selection gradient working with the function spaces $L^p[0,1]$ for $1 \le p \le \infty$ since these spaces contain the strategies in the function-valued game. Since continuous and bounded measurable functions on the compact interval [0, 1] are contained in $L^2[0,1]$, we may use the $L^2$ inner product on $L^2$ functions, denoted by $\langle \,,\rangle$, to compute the selection gradient. Computing the selection gradient amounts to taking the functional derivative of *E*[*f*, *g*] at $g=f$. That is, $\langle \nabla E(f),v\rangle = \left. \frac{d}{dt}\right| _{t=0} E[f,f+tv]$, for any *v* in $L^2[0,1]$. By computing this for arbitrary *v* we find the selection gradient

2.8 $$\begin{aligned} \nabla E(f)(x) = \int _0^x f(y)\,dy - \int _x^1 f(y)\,dy. \end{aligned}$$

The definitions of continuous and bounded measurable strategies (2.4) require that the function *f* be nonnegative. However, in dynamics, there has to be the possibility that the function *f* decreases at some $x\in [0,1]$. If $\nabla E(f)(x)\ge 0$ were true for all $x\in [0,1]$, then the only possible change to *f* would be that it grows. Consequently, the dynamics cannot be restricted to the space of strategies. Therefore, we will consider the adaptive dynamics for functions in the $L^p[0,1]$ spaces for $1\le p\le \infty$. Although this approach does not preserve non-negativity, we can make the dynamics respect the MCA constraint (2.4).

The procedures that are needed to deal with inequality constraints are described by Dieckmann *et al.*^24^. Since only inequality constraints are treated in the context of the current work, we focus on such constraints here. The global inequality constraints are of the form

$$\begin{aligned} w(f)\le 0 \end{aligned}$$

for all *f* under consideration (eg $f \in L^p[0,1]$ for some $1 \le p \le \infty$). Here *w* maps the function space under consideration to $\mathbb {R}$ and is chosen based on the physical constraints in the modeling situation. Starting from a variance-covariance function $U_f(x,y)$, the following transformed variance-covariance function ensures that $w(f)\le 0$ is satsified by the dynamics:

$$\begin{aligned} \sigma ^2_f(x,y)=\int _\Omega \int _\Omega \tilde{P}(x,r)U_f(r,s)\tilde{P}(s,y)\,dr\,ds. \end{aligned}$$

Here, the projection $\tilde{P}$ is defined by the equation

$$\begin{aligned} \tilde{P}(x,y)=\delta _x(y)-\tilde{N}_f(x)\tilde{N}_f(y)H(w(f))H\left( \int _\Omega g_f(z)\tilde{N}_f(z)dz\right) , \end{aligned}$$

where *H* is the Heaviside function (i.e., the indicator function supported on $x\ge 0$) and

$$\begin{aligned} \tilde{N}_f(x)=\frac{N(x)}{\sqrt{\int _\Omega (N(y))^2\,dy}},\quad N(x)=\left. \frac{d}{dt}\right| _{t=0} w(f+t\delta _x). \end{aligned}$$

Global *equality* constraints can be taken into account by removing the factor *H*(*w*(*f*)) from the above expression for $\tilde{P}(x,y).$ Notice that Dieckmann *et al.*^24^ use the opposite sign convention on the inequality constraint, which implies that also *N* has the opposite sign in this presentation.

The selection gradient maps the nonnegative elements of $L^\infty [0,1]$, denoted $\mathcal {L}^\infty _+[0,1]$ into $L^\infty [0,1]$ (that is, not into $\mathcal {L}^\infty _+[0,1]$). Since $L^\infty [0,1]$ is a subspace of $L^2[0,1]$, the inner product of $L^2$ can be used to project the selection gradient onto the subspace of functions that satisfy the MCA constraint. This constraint can be expressed as

2.9 $$\begin{aligned} w(f)\le 0\quad \text { for }\quad w(f)=\int _0^1 (x-1/2)f(x)\,dx. \end{aligned}$$

The projection onto the tangent of the boundary $w(f)=0$ is thus

$$\begin{aligned} P(f) = \frac{\langle f, \nabla \hspace{-0.15em} w\rangle }{\Vert \nabla \hspace{-0.15em} w\Vert ^2} \nabla \hspace{-0.15em} w,\qquad \nabla w(x)=x-\frac{1}{2}. \end{aligned}$$

Here, $\langle \, ,\rangle$ is the $L^2$ inner product on [0, 1], and $\Vert \nabla w\Vert ^2=\langle \nabla w,\nabla w\rangle$. The projection of the selection gradient $\nabla E$ onto the normal direction of $w(f)=0$ is given by

2.10 $$\begin{aligned} P\big (\nabla E(f)\big )(x) = \frac{\int _0^1 (y-1/2)\left( \int _0^y f(z)\,dz-\int _y^1 f(z)\,dz\right) dy}{\int _0^1 (y-1/2)^2 dy}(x-1/2). \end{aligned}$$

Notice that *P* maps any function onto a linear function on $\mathbb {R}$. Removing the component of the selection gradient $\nabla E(f)$ which is normal to $w(f)=0$ is achieved by projection with $1-P$, where 1 is the identity mapping. This ensures that the constraint in (2.9) is respected at all times. These results are consistent with the treatment of global inequality constraints as described by Dieckmann *et al.*^24^, as we show in the Methods §4.1. Applying this variance-covariance function is equivalent to the projection by $1-P$, where *P* is defined in (2.10). The full adaptive dynamics of the function-valued game constrained to MCA$(f)\le \frac{1}{2}$ is given by the initial-value problem

2.11 $$\begin{aligned} \frac{\partial }{\partial t} f = (1-H(w(f))P)\nabla E(f),\qquad \text { with } \left. f\right| _{t=0} = f_0. \end{aligned}$$

Here $f=f(x,t)$ is a one-parameter family of functions, depending on the parameter *t*, mapping $x\in [0,1]$ to $\mathbb {R}$, and *P* is defined by (2.10). We refer to $(1-P)\nabla E$ as the *constrained selection gradient, *whereas $\nabla E$ is the *unconstrained selection gradient. *The unconstrained selection gradient $\nabla E$ is an integral operator with kernel *s*(*x*, *y*) defined by

2.12 $$\begin{aligned} \nabla E(f)(x) = \int _0^1 s(x,y)f(y)\, dy, \quad s(x,y) = \chi _{[0,x)}(y)-\chi _{(x,1]}(y) = {\left\{ \begin{array}{ll} -1, & x<y,\\ 1, & x>y. \end{array}\right. } \end{aligned}$$

The kernel is constant above and below the diagonal $x=y$. It is weakly singular on the diagonal, that is, it is undefined on the set $x=y$ with $x,y\in [0,1]$.

The constrained selection gradient may also be defined as a kernel operator. For this it is convenient to introduce the notation

2.13 $$\begin{aligned} A f(x)= & (1-H(w(f))P) \nabla E f(x) \nonumber \\= & \int _0^x f(y)\,dy -\int _x^1 f(y)\,dy \nonumber \\ & -12 H(w(f)) (x-\tfrac{1}{2})\int _0^1\left( y-\frac{1}{2}\right) \left( \int _0^y f(z)\,dz -\int _y^1 f(z)\,dz\right) dy. \end{aligned}$$

Above, *H* is the Heaviside function, $12 = \langle x-\frac{1}{2},x-\frac{1}{2}\rangle ^{-1}$ is a normalization factor, and *w* is defined in (2.9). In order to define *A* as a kernel operator, recall the integration by parts from equation (4.2), and let *s* be the kernel of $\nabla E$ as in (2.12). As a kernel operator, *A* is then given by

2.14 $$\begin{aligned} \begin{aligned}&Af(x)=\int _0^1 k(x,y)f(y)\,dy,\\ &\text {with}\quad k(x,y) = s(x,y)+12 H(w(f))\left( x-\tfrac{1}{2}\right) \left( y^2-y\right) . \end{aligned} \end{aligned}$$

Notice that if we accept distributions in our theory we may write the mapping *A* as a kernel operator on the gradient:

2.15 $$\begin{aligned} \begin{aligned}&Af(x)=\int _0^1 \sigma ^2(x,y)\nabla E(f)(x)\,dy,\\&\text {with}\quad \sigma ^2(x,y) = \delta _x(y)-12H(w(f))\left( x-\tfrac{1}{2}\right) \left( y-\tfrac{1}{2}\right) . \end{aligned} \end{aligned}$$

Here, we denote the kernel by $\sigma ^2$, since that correctly describes the connection to the adaptive dynamics framework of Dieckmann *et al.*^24^. This notation matches theirs, as can be seen in equation (2.5). In the case ${{\,\textrm{MCA}\,}}(f)<\frac{1}{2}$, the second term of $\sigma ^2$ is left out, that is, $\sigma ^2(x,y)=\delta _x(y)$. This, too, is consistent with the framework by Dieckmann *et al.*^24^. They further remark that the “boundary layer induced by inequality constraints will be very narrow whenever the canonical equation offers a valid description.” Therefore, there is no smooth transition from $\sigma ^2(x,y)=\delta _x(y)$ to $\sigma ^2(x,y)=\delta _x(y)-12(x-\frac{1}{2})(y-\frac{1}{2})$. The change is abrupt.

Our first major result identifies the eigenfunctions and eigenvalues of the system $Af = \lambda f$ for *A* as in (2.14). This is useful for the adaptive dynamics because it will allow us to identify the stationary solutions, namely those that satisfy $\frac{d}{dt}f=Af=0$.

#### Theorem 2.4

Let *A* be defined by (2.11), and fix some $1 \le p \le \infty$. Let *A* act on the elements of $L^p[0,1]$. Then the only solutions to the eigenvalue problem $Af=\lambda f$ are constant functions, and the corresponding eigenvalue $\lambda =0$.

The proof of this theorem is contained in the Methods §4.2. Next, we investigate solutions to the initial value problem for the constrained adaptive dynamics (2.11), with constrained selection gradient (2.13), and with initial data contained in $L^p[0,1]$ for $1 \le p \le \infty$. For such initial data $f_0$, define $\alpha$ as the integral curve of *A* starting at $f_0$

2.16 $$\begin{aligned} \frac{d}{dt} \alpha (t)=A\big (\alpha (t)\big )\quad \text { with }\quad \alpha (0)=f_0. \end{aligned}$$

An important question about the problem (2.16) is whether there exist stationary solutions, that is, functions *f* such that $\frac{d}{dt}f=Af=0.$ Our next result shows that the only stationary solutions are constant functions, which are precisely the Nash equilibria of the game as given in Theorem 2.2.

#### Proposition 2.5

The only solutions to the equation for stationary solutions, $\frac{d}{dt}f=Af=0$, with *A* as in (2.14) and initial data in $L^p[0,1]$ for some $1 \le p \le \infty$ are constant functions.

#### Proof

By Theorem 2.4, the equation $Af=\lambda f$ has only one solution *f* such that *f* is not the zero function, namely *f* being a constant on [0, 1]. The corresponding eigenvalue is $\lambda =0$ and therefore, the equation for stationary solutions is satisfied. $\square$

Observe that if the strategies are normalized, they can be interpreted as probability density functions. It turns out that strategies *f* that satisfy ${{\,\textrm{MCA}\,}}(f)=\frac{1}{2}$ stay normalized during the adaptive dynamics evolution. For any function *f*,

$$\begin{aligned} \int _0^1 P(f)(x)\,dx=0, \end{aligned}$$

since *P*(*f*)(*x*), which is defined in (2.10), is proportional to $x-\frac{1}{2}$. By integration by parts,

2.17 $$\begin{aligned} \int _0^1 \left( \int _0^x f(y)\,dy - \int _x^1 f(y)\,dy\right) \,dx= 2\left( \frac{1}{2}-{{\,\textrm{MCA}\,}}(f)\right) \int _0^1 f(x)\,dx. \end{aligned}$$

That is,

$$\begin{aligned} \text {MCA}(f)=\frac{1}{2}\implies \int _0^1 \nabla E(f)(x)\,dx=0. \end{aligned}$$

Therefore, if the initial data $f_0$ is such that ${{\,\textrm{MCA}\,}}(f_0)=\frac{1}{2}$, and *f* evolves according to

$$\begin{aligned} \frac{\partial f}{\partial t} = (1-P)\nabla E(f) = A f,\end{aligned}$$

then the integral $\int _0^1 f(x)\,dx$ is constant. In other words, the population size is preserved. When *f* represents a probability distribution function it stays normalized during the evolution.

Our next result concerns the behavior of the MCA of solutions to the adaptive dynamical system. The proof of this lemma is contained in the Methods §4.2.

#### Lemma 2.6

Assume that the problem (2.16) with *A* defined by (2.11) admits a local solution, $\alpha _t$, for *t* in some interval *J*. If the solution satisfies $\int _0^1 \alpha _t(x)\,dx\ne 0$ at some $t\in J$, and ${{\,\textrm{MCA}\,}}(\alpha _t)=\frac{1}{2}$ then the time derivative of ${{\,\textrm{MCA}\,}}(\alpha )$ at time *t* vanishes. If $0<{{\,\textrm{MCA}\,}}(\alpha _t)<\frac{1}{2}$, and the solution satisfies $\alpha _t(x)>0$ at some $t\in J$ then ${{\,\textrm{MCA}\,}}(\alpha _t)$ is an increasing function of *t* with growth rate $\frac{d}{dt}{{\,\textrm{MCA}\,}}(\alpha _t)>2(1/2-{{\,\textrm{MCA}\,}}(\alpha _t))^2.$

The lower bound of the above lemma approaches $\frac{1}{2}$ as $t\rightarrow \infty .$ To see this, consider a function *g*(*t*) that grows exactly according to $g'=2(1/2-g)^2$. Solving this yields

$$\begin{aligned} g(t) = \frac{1}{2} -\frac{1}{c_0+2t}. \end{aligned}$$

Here, $c_0$ is a constant of integration. To guarantee $g(0)={{\,\textrm{MCA}\,}}(f_0)$ we solve ${{\,\textrm{MCA}\,}}(f_0)=\frac{1}{2}-\frac{1}{c_0}$, where $f_0$ is the initial data. Since ${{\,\textrm{MCA}\,}}(f_0)<\frac{1}{2}$ we obtain $c_0>0$. For any $t\ge 0$, the MCA is increasing, and

$$\begin{aligned} {{\,\textrm{MCA}\,}}(\alpha _t)>\frac{1}{2} -\frac{1}{c_0+2t}\quad \text { for } t>0, \text { and tends to }\frac{1}{2} \text { as }t \rightarrow \infty . \end{aligned}$$

Lemma 2.6 is useful because it is a key ingredient in the following theorem, which is one of our central results. This result gives the existence and regularity of solutions to the adaptive dynamical system.

#### Theorem 2.7

Fix *p* with $1 \le p \le \infty$. Let *A* be as in (2.11) and $f_0\in L^p[0,1]$. Then the initial value problem (2.16) admits a solution $\alpha :[0,\infty )\rightarrow L^p[0,1]$. If in addition $\int _0^1 f_0(x)\,dx\ne 0$ and ${{\,\textrm{MCA}\,}}(f_0)=\frac{1}{2}$ then ${{\,\textrm{MCA}\,}}(\alpha (t))=\frac{1}{2}$ for all $t>0$. If furthermore $f_0\in L^p[0,1]$ with $p\ge 2$ and ${{\,\textrm{MCA}\,}}(f_0)=\frac{1}{2}$ then the $L^2$ norm of the solution is constant for all $t>0.$ If $f_0\in C^k([0,1])$ then the solution is also $C^k$ at every time.

The proof of Theorem 2.7 is contained in the Methods §4.2. Our next result applies the theorem to show that evolution according to this adaptive dynamical system is beneficial: the solution to the adaptive dynamical system evolves to defeat the initial data. This proof is also contained in the Methods §4.2.

#### Lemma 2.8

Let $f_0\in \mathcal {L}^\infty _+[0,1],$ and assume that $f_0$ is not a constant function. Then, for small *t*, the solution to the adaptive dynamics equation (2.16) defeats $f_0$, or in other words $E[\alpha (t),f_0]>0$.

Our next result shows that if the initial data is not a constant function, then the evolution according to the adaptive dynamical system continues changing indefinitely; it is never constant. This shows that our model could account for the tendency of microbe species to continually evolve towards increasing complexity and phenotypic variability.

#### Proposition 2.9

Assume that the initial condition of the problem (2.16) is such that ${{\,\textrm{MCA}\,}}(f_0)=\frac{1}{2}$ and that $f_0$ is not constant. Then the solution to this problem, $\alpha (t)$, is never the constant function.

#### Proof

If *u* is the constant function, without loss of generality assume $u=1$, then for any function *g* in $L^p[0,1]$ for $1 \le p \le \infty$,

2.18 $$\begin{aligned} E[u,g] = 2\left( \tfrac{1}{2}-{{\,\textrm{MCA}\,}}(g)\right) \int _0^1g(x)\,dx. \end{aligned}$$

Let $\alpha$ be the integral curve of *A* with $\alpha (0)=f_0$. Since $f_0$ is not constant, for sufficiently small $t>0$,

$$\begin{aligned} E[\alpha (t),f_0]>0 \end{aligned}$$

by Lemma 2.8. This is impossible if $\alpha (t)$ is the constant function by equation (2.18), since ${{\,\textrm{MCA}\,}}(f_0)=\frac{1}{2}$. $\square$

Our next result shows that if we begin with a strategy of the function-valued game, and the strategy is strictly positive, then there exists a solution to the adaptive dynamical system that is also a strategy of the function-valued game.

#### Theorem 2.10

Let $f_0$ be either continuous or an element of $L^\infty [0,1]$. Assume that it satisfies (2.4) and $f_0(x)\ge K$ for some $K>0$ and for all $x\in [0,1]$. Then for some $T>0$ there exists a solution $\alpha$ to (2.16) that satisfies (2.4) for $t\in [0,T]$ and is respectively, continuous or an element of $L^\infty [0,1]$ for all $t \in [0,T]$.

#### Proof

First, observe that continuous functions on [0, 1] are all contained in $L^\infty [0,1]$. The problem (2.16) admits a solution $\alpha :[0,\infty )\rightarrow L^\infty [0,1]$ by Theorem 2.7, which is as smooth as the initial data. Thus $\alpha (t)-f_0$ is well-defined in $L^\infty [0,1]$ for all $t\in [0,T]$ for some $T>0.$ Define

$$\begin{aligned} \bar{M} = \sup _{t\in [0,T]} \Vert \alpha (t)-f_0\Vert _\infty = \sup _{t\in [0,T]} \sup _{x\in [0,1]}|\alpha (t)(x)-f_0(x)|. \end{aligned}$$

Then,

$$\begin{aligned} \Vert \alpha (t)-f_0\Vert _\infty =\left\| \int _0^t A\alpha (s)\,ds\right\| _\infty \le t\bar{M}\quad \text { for all } t<T. \end{aligned}$$

This equation shows that if *t* is sufficiently small, then $\alpha (t)$ is sufficiently close to $f_0$ in the supremum norm. Therefore, if $f_0$ is such that $f_0(x)\ge K$ for all *x* and for some $K>0$, we fix a number $\varepsilon$ such that $0<\varepsilon <K$ and then select $T>0$ small enough so that $\alpha (t)(x)\ge \varepsilon$ for all $t\in [0,T]$ and all $x\in [0,1].$ $\square$

As a consequence of Theorem 2.10 and Lemma 2.8, the adaptive dynamical system with initial data given by a strictly positive function-valued strategy evolves to a stronger strategy, in the sense that the evolved strategy defeats the initial strategy.

#### Corollary 2.11

If $f_0$ is not a constant function, and if $f_0(x)\ge K>0$ for all *x*, then the adaptive dynamics evolves to a strategy which defeats $f_0$.

The line of mathematical arguments and proofs, from Theorem 2.4 to Lemma 2.11, characterizes the evolution of species in the game dynamics. All species experience evolution, via the operator *A*, except for the species that have a flat, that is constant, distribution of their competitive abilities. This conclusion aligns not only with the properties of the Nash equilibria^18^, but also with empirical observations of planktonic microbe species^7,16,17^. Here, however, our results are from a dynamical point of view. In other words, we see that the game theoretic equilibria and the dynamical equilibria agree, and both appear to be consistent with empirical observations. According to the findings here and in agreement with the construction of adaptive dynamics in general, the dynamics of each species selects traits that have a competitive advantage against its resident population, as described in Lemma 2.8.

### Evolution towards the equilibrium in the function-valued adaptive dynamics

The stationary points of the adaptive dynamics are specified in Proposition 2.5. Let *u* be a uniform distribution over the unit interval, that is, *u*(*x*) is a positive constant for all *x* in the interval $0\le x\le 1.$ It is a stationary point for the adaptive dynamics as well as an equilibrium strategy in the game. By “reversing time,” we can show that there exist strategies that will evolve to such an equilibrium strategy. The idea is that the following problems are identical: First, consider the forward-time problem

2.19 $$\begin{aligned} \dot{\alpha } = \nabla E(\alpha ), \quad \alpha (0)=\alpha _0, \quad \alpha (T)=u, \end{aligned}$$

where $\alpha _0$ is a strategy such that ${{\,\textrm{MCA}\,}}(\alpha _0)<\frac{1}{2}.$ Then consider the reverse-time initial value problem

$$\begin{aligned} \dot{\alpha } = -\nabla E(\alpha ), \quad \alpha (0)=u, \quad \alpha (T)=\alpha _0. \end{aligned}$$

There is no unknown in solving this initial value problem, but $\alpha _0$ is determined by assigning it $\alpha (T)=\alpha _0$. Compare this to (2.19), in which $\alpha _0$ is unknown. By reversing time, we may solve an equation with unknown stopping time *T* but with known initial data. It remains to show that there exists a $T>0$ such that $\alpha _0=\alpha (T)$ is a strategy such that ${{\,\textrm{MCA}\,}}(\alpha _0)<\frac{1}{2}$. This is however easy to prove using the following facts. Since *u* is a strictly positive function, there are functions in $L^\infty [0,1]$ that satisfy (2.4) with a strict inequality for the MCA that are arbitrarily close to *u* in the $L^\infty$ norm. By Lemma 2.6, such functions have increasing MCAs. Any such function can be used as $\alpha _0$ above to seed the initial value problem.

### Adaptive dynamics of the vector-valued game

Let $\varvec{y}$ be a column vector representing a strategy in the vector-valued game as introduced in §2.1. Then the component $y_j$ of $\varvec{y}$ is the quantity of individuals with competitive ability equal to $\frac{j}{M}$. We define the $(M+1) \times (M+1)$ matrix

2.20 $$\begin{aligned} L= \begin{bmatrix} 0 & -1 & -1 & ... & -1\\ 1 & 0 & -1 & ... & -1\\ 1 & 1 & 0 & ... & -1\\ \vdots & & & & \\ 1 & 1 & 1 & ... & 0 \end{bmatrix}, \end{aligned}$$

We note that in our convention, the indices of a vector in $\mathbb {R}^{M+1}$ are $\varvec{v}= (v_0, v_1, \ldots , v_M)$. We further make the assumptions on $\varvec{y}$ given in (2.1). Then, it is straightforward to compute that the MCA constraint is an equality if and only if $\varvec{y}$ is orthogonal to the vector with components $(j/M-1/2)$. We therefore define the vector

2.21 $$\begin{aligned} \varvec{w}= \begin{bmatrix} -1/2 \\ 1/M - 1/2 \\ \vdots \\ j/M - 1/2 \\ \vdots \\ 1/2 \end{bmatrix}. \end{aligned}$$

We further compute that ${{\,\textrm{MCA}\,}}(\varvec{y}) \ge 0$ is equivalent to $\varvec{w}\cdot \varvec{y}\ge 0$, where $\cdot$ denotes the standard scalar product between vectors. Consequently the projection onto the normal to the set of strategies $\varvec{y}$ satisfying ${{\,\textrm{MCA}\,}}(\varvec{y})=1/2$ is given by projecting onto the span of $\varvec{w}$. For a strategy $\varvec{y}$ this projected vector is given by multiplying $\varvec{y}$ on the left with the matrix

2.22 $$\begin{aligned} P = \frac{\varvec{w}\varvec{w}^T}{\Vert \varvec{w}\Vert ^2}, \end{aligned}$$

where $\Vert \varvec{w}\Vert ^2=\varvec{w}^T\varvec{w}$ is the sum of the squares of the components of $\varvec{w}.$ Then $I-P$, where *I* is the $(M+1) \times (M+1)$ identity matrix, is the projection onto the set of strategies with MCA equal to 1/2. Analogous to the function-valued setting, adaptive dynamics predicts that strategies evolve according to the linear ODE system

2.23 $$\begin{aligned} \frac{d}{dt} \varvec{y}= A \varvec{y}, \quad \varvec{y}(0) = \varvec{y}_0, \quad A\varvec{y}= (I-H(\varvec{w}\cdot \varvec{y}) P)L \varvec{y}. \end{aligned}$$

Above *H* is the Heaviside function, so that $H(\varvec{w}\cdot \varvec{y})=1$ if and only if ${{\,\textrm{MCA}\,}}(\varvec{y})\ge 1/2$, otherwise it is zero. Here $\varvec{y}_0$ is the initial strategy that is assumed to satisfy (2.1) and the MCA constraint (2.3).

For a system of first order ODEs of this type in (2.23), if an eigenvalue $\lambda$ of *A* has multiplicity *r* and *k* linearly independent eigenvectors $\varvec{v}_1,...,\varvec{v}_k$ with $r=k$, then a basis of solutions for this eigenvalue consists of

$$\begin{aligned} e^{\lambda t} \varvec{v}_1, \ldots , e^{\lambda t} \varvec{v}_r.\end{aligned}$$

If $k<r$, then the basis consists of $e^{\lambda t} \varvec{v}_j$ for $j=1, \ldots , k$ together with $r-k$ solutions of the form $e^{\lambda t}p(t)$, where *p* is a polynomial of degree at most $r-k$ with vector coefficients. These vector coefficients are linear combinations of generalized eigenvectors. We recall that a generalized eigenvector for the eigenvalue $\lambda$ is a nonzero vector $\varvec{v}$ such that for some $m \ge 1$, $(\lambda I - A)^m \varvec{v}=0$ but $(\lambda I - A)^{m-1} \varvec{v}\ne 0$. Consequently, in order to determine the solutions of the ODE system (2.23), we must establish properties of the matrices involved in this system and determine their eigenvalues. The projection matrix *P* is symmetric and real, so its eigenvalues are real. Since it is a projection, all its eigenvalues are equal to either 1 or zero. The matrix *L*, which maps $\varvec{y}$ to the unconstrained selection gradient, is an anti-symmetric Toeplitz type matrix. It has purely imaginary eigenvalues (as do all real anti-symmetric square matrices) that occur in pairs of complex conjugates. If there is an odd number of eigenvalues, one of them is zero. To solve the dynamical system, we therefore demonstrate the following results. The proofs of these results are contained in the Methods §4.3.

First we compute the rank of the matrix *L*, which depends on its dimensions.

#### Lemma 2.12

Let *L* denote the $(M+1) \times (M+1)$ skew-symmetric matrix with all entries above the diagonal equal to $-1$, and all entries below the diagonal equal to 1 as shown in (2.20). Then the rank of *L* is $M+1$ when $M+1$ is even, and it is *M* when $M+1$ is odd.

Next we compute the dimension of the kernel of the product $(I-P)L$. Again this also depends on the dimensions of these matrices, noting that they are all square matrices of the same dimensions.

#### Proposition 2.13

Let *L* and *P* be defined in (2.20) and (2.22), respectively, and *I* be the $(M+1) \times (M+1)$ identity matrix. Then we have

$$\begin{aligned} \dim \textrm{Ker}\, (I-P)L = {\left\{ \begin{array}{ll} 2, & M+1\text { odd}\\ 1, & M+1\text { even.} \end{array}\right. } \end{aligned}$$

We then show that the eigenvalues of a product of a projection matrix and a real square anti-symmetric matrix of the same dimensions are always purely imaginary (or zero).

#### Proposition 2.14

Let *Q* be a projection matrix and *S* be a real square anti-symmetric matrix of the same dimensions. Then the eigenvalues of *QS* are contained in $i \mathbb {R}$.

We now apply the preceding results to characterize the eigenvalues of $(I-P)L$ as well as those of *L*.

#### Corollary 2.15

The non-zero eigenvalues of $(I-P)L$ occur in pairs of the form $\pm i b$ for nonzero $b \in \mathbb {R}$. Zero is an eigenvalue of $(I-P)L$ with geometric multiplicity one if $M+1$ is even, and geometric multiplicity 2 if $M+1$ is odd. The non-zero eigenvalues of *L* occur in pairs of the form $\pm i b$ for nonzero $b \in \mathbb {R}$. Zero is an eigenvalue with geometric multiplicity equal to one precisely when $M+1$ is odd.

We now determine a basis for the kernel of $(I-P)L$ in case its dimensions $(M+1) \times (M+1)$ have $M+1$ odd.

#### Proposition 2.16

Assume that $(I-P)L$ is $(M+1)\times (M+1)$ for $M+1$ odd. Then the vectors $\varvec{v}_o$ and $\varvec{v}_e$ with ones in the odd and even components, respectively, and all other entries equal to zero, constitute a basis of the kernel of $(I-P)L$.

We now determine a basis for the kernel of $(I-P)L$ in case its dimensions $(M+1) \times (M+1)$ have $M+1$ even.

#### Proposition 2.17

Assume that $M+1$ is even. Define the vector $\varvec{v}_2$ $=$ (2, 0, 2, 0,..., 2, 0). Then $\varvec{v}_2$ solves $(I-P)L\varvec{v}_2 = (1,1,1,...,1)$ $=\varvec{v}_1$, and $\varvec{v}_1$ is a basis for the kernel of $(I-P)L$. That is, $\varvec{v}_2$ and $\varvec{v}_1$ consitute a Jordan chain for the eigenvalue zero.

The fundamental theorem for linear systems^29^ gives the explicit form of the solution to (2.23). We first give the case when the initial data satisfies the MCA constraint with equality.

#### Theorem 2.18

Assume that the initial data $\varvec{y}_0 \in \mathbb {R}^{M+1}$ satisfies (2.1) and the MCA constraint (2.3) is an equality. Then the solution to the adaptive dynamics (2.23) with initial data $\varvec{y}_0$ is

$$\begin{aligned} \varvec{y}(t) = Q B Q^{-1} \varvec{y}_0.\end{aligned}$$

The matrix *B* is a real block-diagonal square matrix, and *Q* is real and invertible. These matrices both have dimensions $(M+1) \times (M+1)$. The columns of *Q* are the generalized eigenvectors of $(I-P)L$ ordered in the following way: Let *m* be the algebraic multiplicity of the eigenvalue $\lambda =0$, and let $\varvec{v}_1,\varvec{v}_2,...,\varvec{v}_m$ be a set of generalized eigenvectors for $\lambda =0$. Non-zero eigenvalues occur in conjugate pairs $\lambda =i\beta _j$, $\bar{\lambda }=-i\beta _j$, for $j=m+1,m+2,...,\ell$. If $\varvec{u}_j+i\varvec{w}_j$ is a generalized eigenvector to $\lambda =i\beta _j$, where $\varvec{u}_j$ and $\varvec{w}_j$ are the real and imaginary part of the generalized eigenvector, then a basis for $\mathbb {R}^{M+1}$ and the columns of *Q* are given by

$$\begin{aligned} \varvec{v}_1,\varvec{v}_2,...,\varvec{v}_m, \varvec{u}_{m+1},\varvec{w}_{m+1},...,\varvec{u}_\ell ,\varvec{w}_\ell . \end{aligned}$$

The first generalized eigenvectors are given by Proposition 2.16 if $M+1$ is odd or Proposition 2.17 if $M+1$ is even. Correspondingly, if $M+1$ is odd then $m\ge 3$ and is odd, and if $M+1$ is even then $m\ge 2$ and is even. The matrix *B* consists of blocks along the diagonal corresponding to the eigenvalues, ordered like the above basis. Each block $B_j$ corresponding to the zero eigenvalue, 0, is of the form

$$\begin{aligned} \begin{bmatrix} 1 & t & \frac{t^2}{2} & \dots & \frac{t^{k-1}}{(k-1)!} \\ & 1 & t & \dots & \\ & & 1 & \ddots & \\ & & & \ddots & t\\ & & & & 1 \end{bmatrix} \end{aligned}$$

for some $k \ge 1$, and such that the sizes of these blocks add up to the algebraic multiplicity of $\lambda =0$. All components below the diagonal are zero. For an eigenvalue $i \beta _j \ne 0$, let

$$\begin{aligned} R_j = \begin{bmatrix} \cos (\beta _j t) & -\sin (\beta _j t) \\ \sin (\beta _j t) & \cos (\beta _j t) \end{bmatrix}. \end{aligned}$$

A block $B_j$ corresponding to $\pm i \beta _j$ is of the form

$$\begin{aligned} \begin{bmatrix} R_j & tR_j & \frac{t^2}{2}R_j & \ldots & \frac{t^{k-1}}{(k-1)!}R_j \\ & R_j & tR_j & \ldots & \\ & & \ddots & \ddots & \\ & & & R_j & tR_j \\ & & & & R_j \end{bmatrix} \end{aligned}$$

for some $k \ge 1$. All components below the diagonal blocks are zero.

#### Proof

The form of the solution follows immediately from the fundamental theorem for linear systems and the real Jordan form^29^ of the matrix $(I-P)L$, noting that Propositions 2.13–2.16 apply to this matrix. We note that the algebraic multiplicity is greater than or equal to the geometric multiplicity. Thus when $M+1$ is even, since the blocks corresponding to nonzero eigenvalues are all even-dimensional, the block corresponding to the zero eigenvalue must also be even-dimensional. Since the geometric multiplicity of the zero eigenvalue is one, this shows that its algebraic multiplicity is at least two. When $M+1$ is odd, the geometric multiplicity of the zero eigenvalue is two. Since the blocks corresponding to the non-zero eigenvalues are all even-dimensional, the block corresponding to the zero eigenvalue must be odd dimensional. Therefore the algebraic multiplicity of the zero eigenvalue is at least 3. $\square$

The same arguments, together with Lemma 2.12 and Corollary 2.15 gives the solution in case the initial data has MCA strictly less than 1/2; the details of this proof are contained in the Methods §4.3.

#### Theorem 2.19

Assume that the initial data $\varvec{y}_0 \in \mathbb {R}^{M+1}$ satisfies (2.1) and the MCA constraint (2.3) is a strict inequality. Then the solution to the adaptive dynamics (2.23) with initial data $\varvec{y}_0$ is

$$\begin{aligned} \varvec{y}(t) = Q B Q^{-1} \varvec{y}_0.\end{aligned}$$

If $M+1$ is even, then the $(M+1) \times (M+1)$ invertible matrix *Q* has columns given by the real and imaginary parts of the eigenvectors and generalized eigenvectors of *L*. The matrix *B* consists of blocks along the diagonal corresponding to the eigenvalues of *L*. These blocks are of the same type as in Theorem 2.18. If $M+1$ is odd, then one block is 1, that is a $1\times 1$ block corresponding to the zero eigenvalue, which has algebraic and geometric multiplicity equal to one. The remaining blocks correspond to the eigenvalues $\pm i\beta _j$ for real $\beta _j\ne 0$.

It follows from the preceding two theorems that the solution to (2.23) is of the form

$$\begin{aligned} \varvec{c}_0(t) + \sum \varvec{a}_k(t) \cos (\beta _k t) + \varvec{b}_k (t) \sin (\beta _k t). \end{aligned}$$

Here, $\varvec{c}_0(t)$, $\varvec{a}_k(t)$, and $\varvec{b}_k(t)$ are polynomials of the variable *t* with vector-valued coefficients, which are linear combinations of the generalized eigenvectors of the eigenvalues 0 and $\pm i\beta _k$, respectively. In case the MCA constraint is satisfied with a strict inequality, and $M+1$ is even, then $\varvec{c}_0$ vanishes. In the case of Theorem 2.19 when the initial data satisfies the MCA constraint with a strict inequality, Proposition 2.20 below shows that both the MCA and the sum of the components of the solution are increasing functions. The proof is contained in Methods §4.3.

#### Proposition 2.20

Let $\varvec{y}$ be a solution to $\dot{\varvec{y}}=L\varvec{y}$ with initial data $\varvec{y}_0$ satisfying (2.1) and ${{\,\textrm{MCA}\,}}(\varvec{y}_0)<\frac{1}{2}$. Then ${{\,\textrm{MCA}\,}}(\varvec{y})$ and $\sum _j y_j$ are increasing functions of time at all times for which the solution satisfies ${{\,\textrm{MCA}\,}}(\varvec{y})<1/2$, $y_j\ge 0$ for all *j*, and $\sum _j y_j>0$.

Similar to the function-valued adaptive dynamics, in the vector-valued adaptive dynamics we prove that the stationary solutions are precisely the equilibrium strategies of the associated vector-valued game as introduced in §2.1.

#### Corollary 2.21

The stationary solutions of the vector-valued game adaptive dynamics (2.23) with initial data satisfying (2.1) and the MCA constraint (2.3) are precisely the equilibrium strategies of the game.

#### Proof

A stationary solution must satisfy ${\dot{\varvec{y}}} = 0, \quad \varvec{y}(0) = \varvec{y}_0$. Thus, it will be equal to the initial data for all time. It therefore follows from Proposition 2.20 that for initial data with ${{\,\textrm{MCA}\,}}(\varvec{y}_0) < 1/2$, it cannot be a stationary solution. We are therefore left with the case when the initial data satisfies the MCA constraint with equality. Since a polynomial cannot be identically equal to a nonconstant trigonometric function, the polynomial term $\varvec{c}_0(t)$ in the solution must be constant. Similarly, all of the trigonometric terms must cancel in order to remain constant. The polynomial term arises from the 0 eigenvalue of $(I-P)L$ and its eigenvectors together with its generalized eigenvectors. The constant term in $\varvec{c}_0(t)$ is a linear combination of the eigenvectors, whereas any non-constant terms in $\varvec{c}_0(t)$ arise from the generalized eigenvectors. The eigenvectors of $(I-P)L$ for the eigenvalue 0 are given in Propositions 2.16 and 2.17. We note that the span of these eigenvectors consists precisely of the equilibrium strategies of the vector-valued game. $\square$

### Evolution to an equilibrium strategy in the vector-valued adaptive dynamics

The equilibrium strategies for the discrete game are precisely the stationary points of the adaptive dynamics. Consequently, they remain unchanged by the evolution according to the adaptive dynamics. It may not be immediately apparent that there exist strategies that will evolve to an equilibrium strategy for the discrete game. To demonstrate the existence of such strategies, consider the problem of finding $\varvec{y}_0$ such that

2.24 $$\begin{aligned} {\dot{\varvec{y}}} = L \varvec{y}, \quad \varvec{y}(0)=\varvec{y}_0, \quad \varvec{y}(T)=(1,1,\ldots , 1). \end{aligned}$$

By reversing the time variable, this is equivalent to solving the initial value problem: ${\dot{\varvec{y}}} = - L \varvec{y}$, $\varvec{y}(0) = (1, 1, \ldots , 1)$, and $\varvec{y}(T) = \varvec{y}_0$. In solving this initial value problem, there is no unknown. Instead, we determine $\varvec{y}_0$ by assigning it $\varvec{y}(T)=\varvec{y}_0$. Whenever $\varvec{y}(0)$ has strictly positive elements there is always a $T>0$ such that the problem is solvable, and such that $\varvec{y}(T)$ also has positive elements. Since *T* can be chosen freely, as long as $\varvec{y}(T)$ has non-negative elements, there is a one-parameter family of initial values leading to the stationary solution. The requirement that $\varvec{y}(T)$ has non-negative elements typically implies that *T* cannot be very large, but maybe more importantly, *T* can be arbitrarily small. Although the equilibrium strategies of the game are stationary points for the adaptive dynamics, they are not stable. In §2.9 we show that any perturbation of the stationary solution unsettles the system.

### Branching

Given the linearity of the evolution equation ${\dot{\varvec{y}}}=A\varvec{y}$ it is tempting to imagine that a species which initially has $y_j>0$ for all *j* can be “split” into two species, one of which is $\varvec{a} = (a, a, a, \ldots , a)$ for $a=\min \{y_j\}$. This $\varvec{a}$ species is constant under the adaptive dynamics. Assume that ${{\,\textrm{MCA}\,}}(\varvec{y}_0) = 1/2$. Then we have $\varvec{y}(t)= \varvec{a} + \varvec{v}(t)$, where $v_j\ge 0$, for all $j=0,1,...,M$. Since ${{\,\textrm{MCA}\,}}(\varvec{a}) = 1/2$, and ${{\,\textrm{MCA}\,}}(\varvec{y}_0) = 1/2$, the initial data $\varvec{v}_0$ also satisfies ${{\,\textrm{MCA}\,}}(\varvec{v}_0)=\frac{1}{2}$. However, if $\varvec{v}_0$ is not a stationary solution then $\varvec{y}$ evolves, and at a later time $t>0$ it could be better than $\varvec{y}_0$ in the sense that $E[\varvec{y}(t),\varvec{y}_0]>0$. It could also happen that $\varvec{v}$ evolves in such a way that $\sum v_j$ decreases, causing the population $\sum y_j = \sum a+v_j$ to shrink. So, although the $\varvec{a}$ subspecies has constant population, the other part of the species does not have this guarantee. In fact, it could occur that some $v_j$ become negative. So, there is no way to see that a stable subspecies can safeguard “the whole of the species” from neither mutation nor from attrition.

**Fig. 1:**
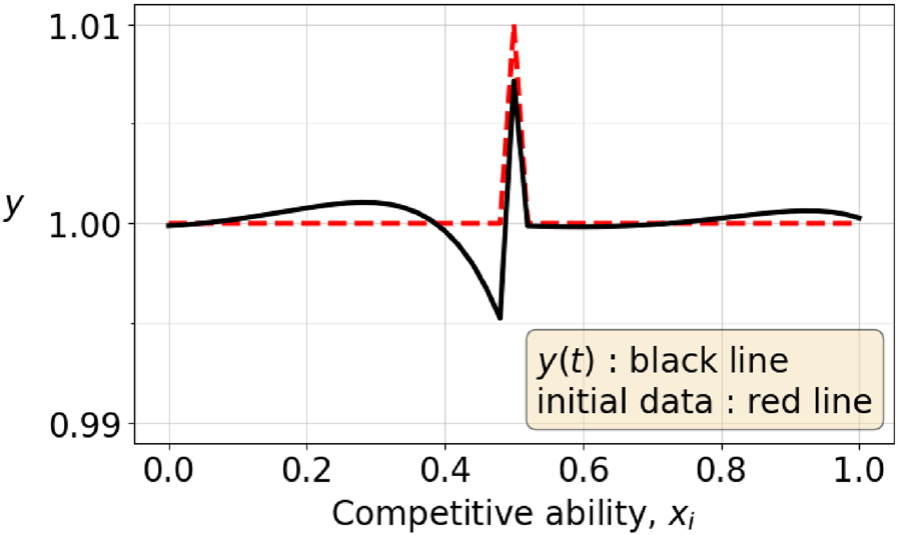
This figure shows an initial strategy, the dashed line, which is an equilibrium strategy $(1, 1, \ldots , 1)$ that has been perturbed by $+0.01$ at its midpoint. After some time, the strategy has evolved according to the black, full line.

**Fig. 2:**
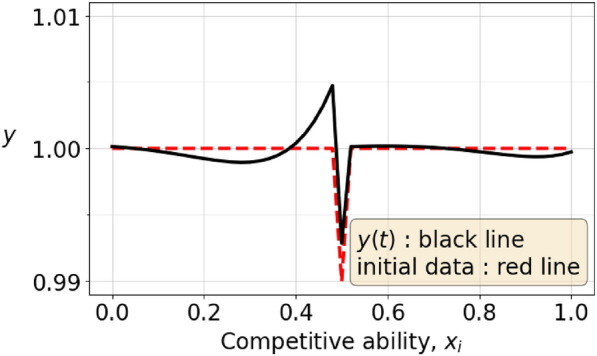
This figure shows an initial strategy, the dashed line, which is an equilibrium strategy $(1, 1, \ldots , 1)$ that has been perturbed by $-0.01$ at its midpoint. After some time, the strategy has evolved according to the black, full line.

We explore this possibility of *branching* by perturbing the equilibrium strategy $(1, 1, \ldots , 1)$ (with 51 sample points $k=0,1,...,50$, so $M=50$) by a small amount at its midpoint. The perturbed strategy $\varvec{y}$ has $y_k=1$ for all *k* except $k=25$, with either $y_{25}=0.99$ or $y_{25}=1.01$. Figure 1 shows the perturbed strategy with $y_{25}=1.01$ as a red line and the resulting branch as the black line, whereas figure 2 shows the the perturbed strategy with $y_{25}=0.99$ as a red line and the corresponding branching strategy in black. The evolved strategies in the two cases are very similar. They are mirror images of each other when mirrored through the equilibrium strategy $(1, 1, \ldots , 1)$.

The results in Figures 1 and 2 can be understood from the linearity of the system (2.23) and the explicit form of the solution depending on the initial data given in Theorems 2.18 and 2.19. Thus a small perturbation $\pm 0.01 \varvec{e}_{26}$, of the initial data $(1, 1, \ldots , 1)$ with $\varvec{e}_{26}$ the $26^{th}$ standard unit vector in $\mathbb {R}^{51}$ drives the evolution in opposite directions for the two opposite signs.

## Discussion

A key feature of our games is the linearity of the payoff functions in their definitions. Linear payoff functions are not applicable in the classification theory that Geritz *et al.*^27^ established for adaptive dynamics. Indeed, a convergence-stable stationary point in the adaptive dynamics evolution is such that (i) its second derivative with respect to the mutant’s strategy is positive and (ii) its second derivative with respect to the resident population’s strategy is larger than the second derivative with respect to the mutant’s strategy. If the payoff is a linear function of the mutant’s strategy, then according to Geritz *et al.*^27^ “once the singular strategy has been established, all mutations are neutral.” Even though this conclusion is reasonable, our results show that the absence of dynamics is a unique feature of the equilibrium strategies of the game. A stationary point cannot be attractive in a linear game, but considering that mutations are random in theory it can be argued that branching is possible in the game. Since the adaptive dynamics setting is a deterministic approximation to a mutation process, which is random, the underlying model assumes that the traits of a species are developing randomly. Thus, the strategies in the current work can be thought of as approximations to traits that are in fact less predictable. From this point of view, we can expect that unstable or neutral stationary points in linear adaptive dynamics are idealizations, and then it would be reasonable to ask what happens if the stationary solutions are perturbed.

In both the function-valued and vector-valued games, it is not clear how to interpret strategies that assume negative values. For this reason, we assume that the initial data is non-negative. For certain initial conditions, the adaptive dynamics may immediately result in either a function *f* that assumes negative values or a vector $\varvec{y}$ that has some component $y_j < 0$. In particular, this can occur if the initial data $f_0$ vanishes at some points in [0, 1] or the initial data $\varvec{y}_0$ has some components $y_j = 0$. To compare this phenomenon for the function- and vector-valued games, we consider samples of $f_0(x)=(x-\frac{1}{2})^2$ at points $x_j=j/M$ with $0\le j\le M$ for some integer $M>2$. Then $f_0$ is positive at the $x_j$ which is closest to (but not equal to) $\frac{1}{2}$. If $M=6$, then $f_0(x_3)=f_0(1/2)=0$, and $f_0(x_4)=1/36.$ Thus, the Newton forward integration would work for small step sizes, since

$$\begin{aligned} f_0(x_4)+\varepsilon (1-P)\nabla E(f_0)(x_4)= \left( \frac{1}{6}\right) ^2+\varepsilon \left( \frac{2}{3}\left( \frac{1}{6}\right) ^3-\frac{1}{10}\frac{1}{6}\right) =\frac{1}{36}-\frac{11\varepsilon }{810}. \end{aligned}$$

**Fig. 3:**
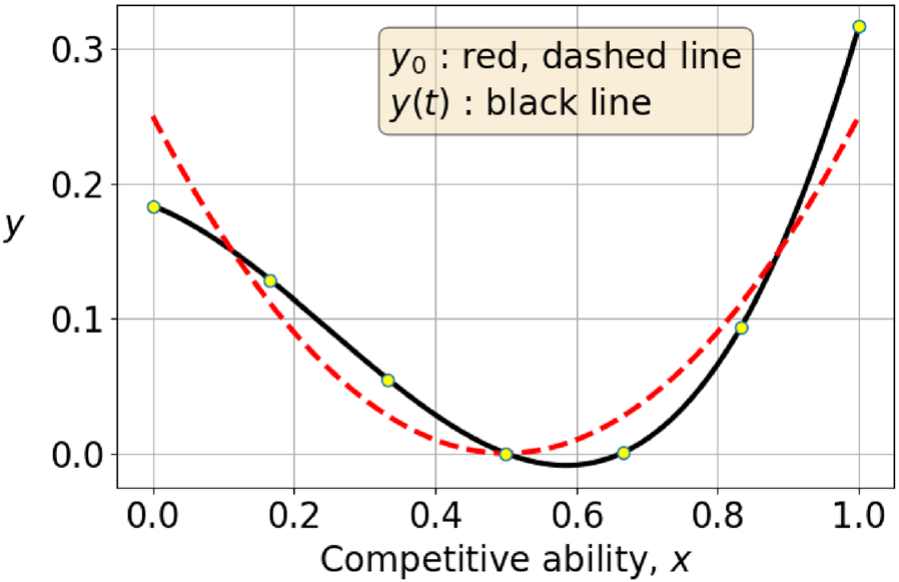
In this figure, the red, dashed line is an initial function $f_0=(x-\frac{1}{2})^2$. The black, full line shows the evolved function just a small time after initial time. The samples of a function-valued strategy, shows as dots here, may be positive even if the underlying function would not remain non-negative during the evolution. This shows one possible loss-of-information in the vector-valued adaptive dynamics as compared to the function-valued adaptive dynamics.

Then, the $f_0(x_4)+\varepsilon (1-P)\nabla E(f_0)(x_4)>0$ for small $\varepsilon$. This is visualized in figure 3, where the red, dashed line is $f_0+\varepsilon (1-P)\nabla E(f_0)$ with $\varepsilon =2$. For *x* larger than $\frac{1}{2}$ but still sufficiently close to $\frac{1}{2}$, the selection gradient changes the strategy to negative values, shown in figure 3. The red dots in the same figure show the samples of the evolved strategy at $x_0,x_1,...,x_6$, and none of them are negative. It should be noted that whereas the initial condition $f_0(x)=(x-\frac{1}{2})^2$ remains a strategy for short times in the discrete game, it may eventually also evolve to have some negative components. We also expect that there are strategies that for both the vector- and function-valued game immediately evolve towards negative values and thus do not represent strategies in the games.

**Fig. 4:**
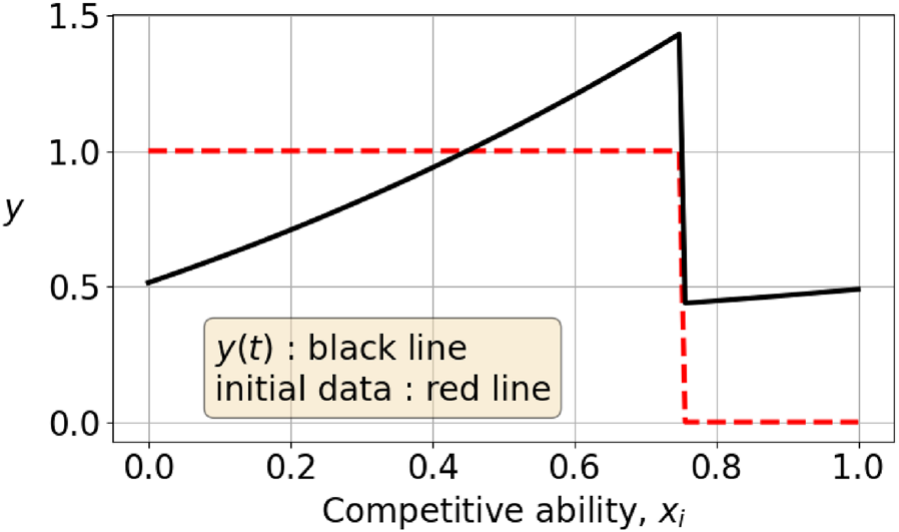
In this figure, the strategy $\varvec{y}$ is shown as the black, full line. It is the evolution of the initial condition which is shown as the red, dashed line. The initial condition satisfies ${{\,\textrm{MCA}\,}}(\varvec{y})<\frac{1}{2}$.

**Fig.5:**
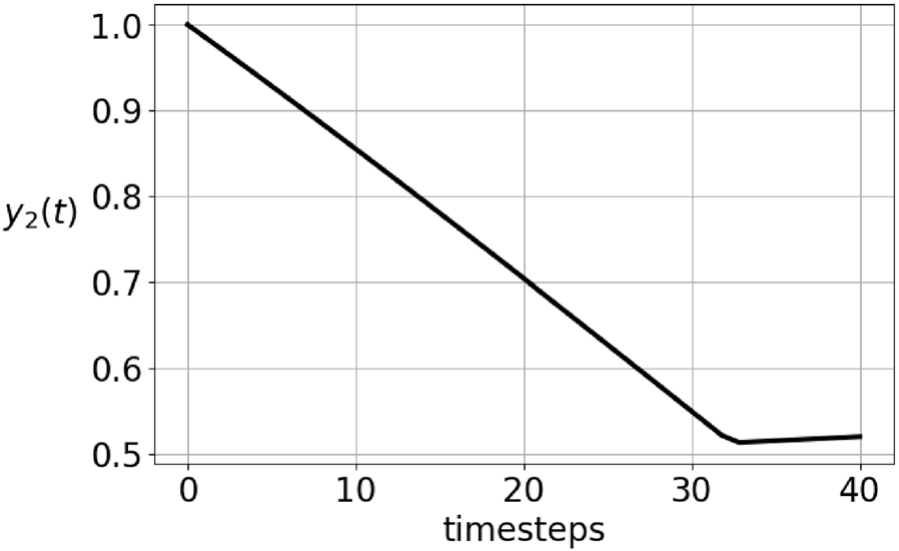
This figure shows the second component of $\varvec{y}$ at timesteps *t* from the initial time to 40 timesteps. The strategy is the same as in Figure 4. It starts with ${{\,\textrm{MCA}\,}}(\varvec{y})<\frac{1}{2}$, but just after 30 timesteps, ${{\,\textrm{MCA}\,}}(\varvec{y})=\frac{1}{2}$.

**Fig. 6:**
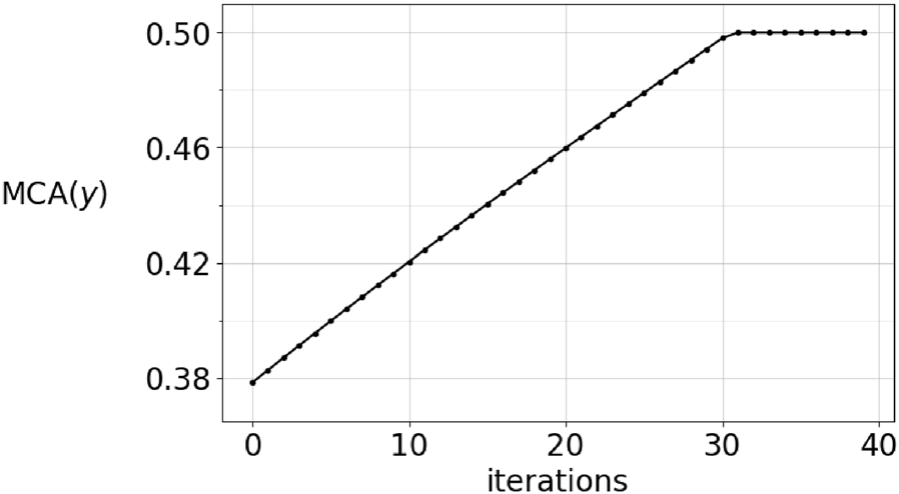
This figure shows the evolution of ${{\,\textrm{MCA}\,}}(\varvec{y})$ with integration step size $\varepsilon =0.02$. The strategy is the same as in Figure 4. Each dot is a sample point. Despite the larger step size, the behavior is not different from Figure 7.

**Fig. 7:**
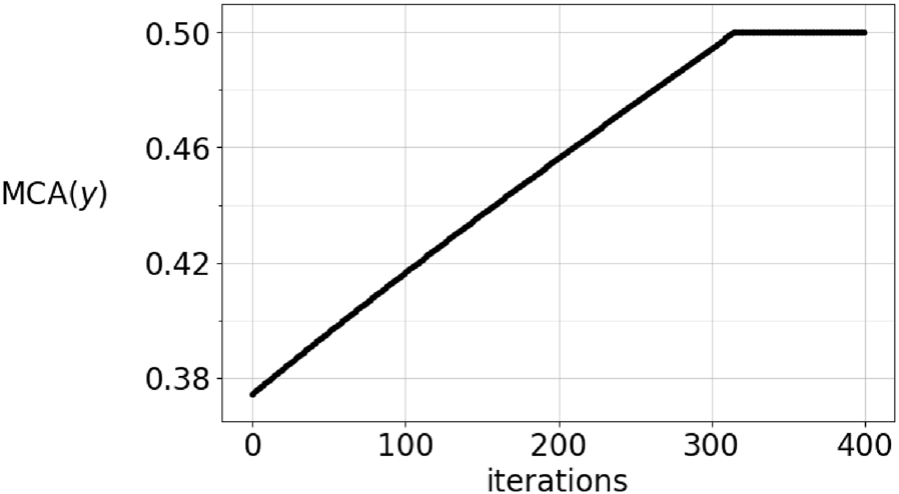
This figure shows the evolution of ${{\,\textrm{MCA}\,}}(\varvec{y})$, where $\varvec{y}$ is the same strategy as in Figure 6, but it is computed with integration step size $\varepsilon =0.002$. This line is also dotted, but the dots are very dense. The results should therefore be reliable regardless of the step size.

If the MCA constraint is an equality, then in both games the projection $I-P$ is applied, whereas when it is a strict inequality, then we do not project. This results in a discontinuity in the formulation of the dynamical system. To see how this affects the solution of the adaptive dynamics in the vector-valued case, Figure 4 shows an initial data with low mean competitive ability and the corresponding solution after 500 timesteps. The solution’s MCA grows and at $t\approx 300$ timesteps, there is a sharp change of direction of the components, as seen in figure 5. This is a result of the dynamics changing equation from ${\dot{\varvec{y}}} = L \varvec{y}$ to ${\dot{\varvec{y}}} = (I-P)L \varvec{y}$. The ${{\,\textrm{MCA}\,}}$ transitions abruptly to the constant ${{\,\textrm{MCA}\,}}(\varvec{y})=\frac{1}{2}$, and as can be seen in Figures 6 and 7, this is not an artefact of the integration step size. In Figure 6, the stepsize $\varepsilon$ in the integration method is 0.02 whereas it is 0.0002 in Figure 7. Consequently, the point where ${{\,\textrm{MCA}\,}}(\varvec{y})=\frac{1}{2}$ is reached after 30 iterations and 3000 iterations, respectively. This result is independent of the sampling frequency $\frac{1}{M}$.

The ${{\,\textrm{MCA}\,}}$ of a function-valued strategy is an increasing function of time, whenever ${{\,\textrm{MCA}\,}}(f_0)<\frac{1}{2}$, according to Lemma 2.6. The lower bound is expressed in the inequality ${{\,\textrm{MCA}\,}}(\alpha _t)>\frac{1}{2}-\frac{1}{c_0+2t}$, $t>0$, where $c_0$ is determined by the initial value ${{\,\textrm{MCA}\,}}(f_0)=\frac{1}{2}-\frac{1}{c_0}.$ How close to being an equality is this? The numerical results in Figures 6 and 7 reaches ${{\,\textrm{MCA}\,}}(\alpha _t)=\frac{1}{2}$ quickly even if the MCA of the initial condition is strictly less than $\frac{1}{2}$. The lower bound $g(t)=\frac{1}{2}-\frac{1}{c_0+2t}$, however, does not reach ${{\,\textrm{MCA}\,}}(g)=\frac{1}{2}$ at finite time, so the real MCA is significantly larger than this lower bound.

The distribution of competitive ability within a species can be the result of either standing genetic variation or arise from a monoclonal population where the phenotype for each individual (i.e. the competitive ability) has a stochastic element and follows a certain distribution. The latter interpretation is the standard one within adaptive dynamics, i.e. a monoclonal population with a certain function-valued trait (the distribution of competitive abilities) is invaded (or not) by a mutant with slightly different distribution^20^. However, the former interpretation is also reasonable, but here change in the resident population is possible not only via invasion, but also acquired mutations that alter the distribution of competitive abilities. How this interpretation should be treated from a mathematical perspective is not clear, since the standard adaptive dynamics framework cannot capture intraspecies genetic variability.

To investigate how well the games and the predictions of adaptive dynamics fit with empirical observations, one could conduct experiments involving competition between strains or species. In order to test the predictions of the model, we suggest that the following conditions should be met. (1) Two or more species (or strains) that compete for the same resources should be studied simultaneously. They need to be asexual and they should ideally reside in a relatively homogeneous environment such that spatial or temporal separation is unlikely. Likewise, each member of a species should be able to compete with any member of any other species. There should not be any “protected groups” in the ecological system. (2) The competitive ability of the species needs to be observable. Moreover, it needs to be quantitative; the competitive ability of an individual should be represented by a number. It is possible that the competitive ability is a compound ability (consisting of several abilities) as long as the individuals can be ordered from low competitive ability to high competitive ability. (3) Every species’ mean competitive ability should be bounded above by the same value. (4) The experiment has to run for a sufficiently long time so that evolution can be observed. This allows for observations of the dynamics of evolution. (5) Mutations that affect the considered trait have to be rare enough so that genetic variation does not arise during the experiment, which could confound the interpretation of the results. The results of such a study could reveal whether or not the theory presented in this paper can explain the evolutionary dynamics of certain species. In particular our theory could be a helpful tool in biogeography, where it is recommended that manipulative experiments and temporal data sets are to be combined with theoretical models in order to explore the diversity and composition of species^30^.

Should our theoretical results be further validated through experiments, it may elucidate why the vast amount of microbial species is seemingly a paradox from the theoretical point of view^11^. Models in competition theory have described that a number of species is limited by the number of key resources. This should raise the concern that such models fail to describe the ecology of microbes. In this work, we have aimed at presenting a model that allows a vast number of species to simultaneously co-exist and compete for survival. As it turns out, the adaptive dynamics applied to these games is constantly changing the composition of a species for nearly all species. The only exception to this are the species characterized by the equilibrium strategies of the game. These equilibrium strategies are the only stationary points of the adaptive dynamics. However, there is no stability in the dynamics in the sense that any perturbation of a stationary point will unsettle the dynamics. These results align well with the idea that evolution does not stabilize and moreover, it does not put a restriction on the number of species. Mechanisms such as genetic drift will make sure that any species accrues DNA changes. Even “living fossils” such as coelacanths are never static^31^. This also fits with “biology’s first law” the tendency for diversity and complexity to increase in evolving systems^32^.

## Methods

In the adaptive dynamics framework, the selection gradient is the driving force behind the evolution of the function-valued traits. The mathematical properties of the selection gradient predict the behavior of the evolution on both long and short timescales.

### Results on the constrained and unconstrained selection gradients for the function-valued adaptive dynamics

A function *f* from [0, 1] to $\mathbb {R}$ is mapped by the (constrained or unconstrained) selection gradient onto a function with properties that depend on the original properties of *f*. We calculate basic properties of the unconstrained selection gradient. It is a linear:

$$\begin{aligned} \nabla E(f+g)(x) = \nabla E(f)(x) + \nabla E(g)(x). \end{aligned}$$

Moreover, $\nabla E(f)$ is a non-decreasing function, which is clear once we rewrite (2.8) as

4.1 $$\begin{aligned} \nabla E(f)(x) = 2\int _0^x f(y)\,dy - \int _0^1 f(y)\,dy. \end{aligned}$$

Next, we derive the constrained selection gradient using the framework that is described by Dieckmann *et al.*^24^. We refer to their work or to Section 2.5 for the notations $\tilde{N}_f$, $g_f$ and $\tilde{P}$. First, observe that

$$\begin{aligned} H\left( \int _\Omega g_f(x)\tilde{N}_f(x)dx\right) = H\left( \sqrt{12}\int _0^1 \left( x-\frac{1}{2}\right) \left( \int _0^x f(y)\,dy-\int _x^1 f(y)\,dy\right) dx\right) . \end{aligned}$$

By integration by parts

4.2 $$\begin{aligned} \int _0^1 (x-\tfrac{1}{2})\left( \int _0^x f(y)\,dy-\int _x^1 f(y)\,dy\right) dx = -\int _0^1 (x^2-x)f(x)\,dx\ge 0. \end{aligned}$$

Therefore, $$H(\int _\Omega g_f(x)\tilde{N}_f(x)dx)=1$$. Then

$$\begin{aligned} \tilde{P}(x,y)=\delta _x(y)-H(w(f))12(x-\tfrac{1}{2})(y-\tfrac{1}{2}). \end{aligned}$$

Here, *w* is defined in (2.9). Consequently

$$\begin{aligned} \sigma ^2_f(x,y)&=\int _0^1\left( U_f(x,s)-12H(w(f))(x-\tfrac{1}{2})\int _0^1(r-\tfrac{1}{2})U_f(r,s)\,dr\right) \tilde{P}_f(s,y)ds\\&=U_f(x,y)-12H(w(f))(x-\tfrac{1}{2})\int _0^1(r-\tfrac{1}{2})U_f(r,y)\,dr -12H(w(f))(y-\tfrac{1}{2})\int _0^1(s-\tfrac{1}{2})U_f(x,s)\,ds\\&+12^2H(w(f))^2(x-\tfrac{1}{2})(y-\tfrac{1}{2})\int _0^1\int _0^1(r-\tfrac{1}{2})U_f(r,s)\,dr (s-\tfrac{1}{2})ds. \end{aligned}$$

In the special case $U_f(x,y)=\delta _x(y)$ we obtain

$$\begin{aligned} \sigma ^2_f(x,y)=\delta _x(y)-12H(w(f))(x-\tfrac{1}{2})(y-\tfrac{1}{2}) -12H(w(f))(x-\tfrac{1}{2})(y-\tfrac{1}{2})\\ +12^2H(w(f))(x-\tfrac{1}{2})(y-\tfrac{1}{2})\underbrace{\int _0^1(s-\tfrac{1}{2})^2ds}_{=1/12} =\delta _x(y)-12(x-\tfrac{1}{2})(y-\tfrac{1}{2}) H(w(f)). \end{aligned}$$

That is, we recover the kernel of the adaptive dynamics operator (2.15).

The following lemma shows that both the constrained (2.11) and the unconstrained (2.8) selection gradient enjoy certain mapping properties.

#### Lemma 4.1

The constrained and unconstrained selection gradients, *A* and $\nabla E$, respectively defined in (2.13) and (2.8), are bounded operators from $L^p[0,1]$ into $L^p[0,1]$ for all $1\le p\le \infty$. Moreover, they satisfy the following mapping properties:

4.3 $$\begin{aligned} L^p[0,1]&\mapsto W^{1,p}[0,1],\quad 1\le p\le \infty , \end{aligned}$$

4.4 $$\begin{aligned} C^k[0,1]&\mapsto C^{k+1}[0,1], \quad 0 \le k < \infty . \end{aligned}$$

Here, $W^{1,p}[0,1]$ is the Sobolev space of functions in $L^p[0,1]$ such that their (weak) derivatives of first order are contained in $L^p[0,1]$.

#### Proof

The key to this proof is Hölder’s inequality, $\Vert fg\Vert _1\le \Vert f\Vert _p\Vert g\Vert _{p'}$, on $f\in L^p[0,1]$ and $g\in L^{p'}[0,1]$, where $1/p+1/p'=1$. If $p=\infty$ then $p'=1$. Consider first the unconstrained selection gradient:

$$\begin{aligned} \left| \nabla E(f)(x)\right| =\left| \int _0^x f(y)\,dy -\int _x^1 f(y)\,dy\right| = \left| \int _0^1 (\chi _{[0,x]}(y)-\chi _{[x,1]}(y))f(y)\,dy\right| . \end{aligned}$$

Then apply Hölder’s inequality:

$$\begin{aligned} \left| \int _0^1 (\chi _{[0,x]}(y)-\chi _{[x,1]}(y))f(y)\,dy\right| \le \Vert \chi _{[0,x]}-\chi _{[x,1]}\Vert _{p'}\Vert f\Vert _p \\ = \Vert f\Vert _p\left( \int _0^1|\chi _{[0,x]}(y)-\chi _{[x,1]}(y)|^{p'}dy\right) ^{1/p'}=\Vert f\Vert _p\left( \int _0^1\,dy\right) ^{1/p'}=\Vert f\Vert _p. \end{aligned}$$

Therefore, $|\nabla E(f)(x)|^p \le \Vert f\Vert _p^p$ which implies

4.5 $$\begin{aligned} \int _0^1|\nabla E(f)(x)|^p dx\le \Vert f\Vert _p^p. \end{aligned}$$

This is true also in the case $p=1$, since $\Vert \chi _{[0,x]}-\chi _{[x,1]}\Vert _\infty \Vert f\Vert _1=\Vert f\Vert _1.$ In case $p=\infty$ we have

$$\begin{aligned} |\nabla E f(x)| \le \int _0 ^x ||f||_\infty + \int _x ^1 ||f||_\infty = ||f||_\infty \implies ||\nabla E f||_\infty \le ||f||_\infty .\end{aligned}$$

In order to show the same type of estimate on the constrained selection gradient we compute

$$\begin{aligned} \Vert P\big (\nabla E(f)\big )\Vert _p = \left( \int _0^1|x-\tfrac{1}{2}|^{p}dx\left| 12\int _0^1\big (y-\tfrac{1}{2}\big )\big (\nabla E(f)\big )dy\right| ^p\right) ^{1/p}\\ =12\Vert x-\tfrac{1}{2}\Vert _p\left| \int _0^1\big (y-\tfrac{1}{2}\big )\big (\nabla E(f)\big )dy\right| \\ \le 12\Vert x-\tfrac{1}{2}\Vert _p \Vert x-\tfrac{1}{2}\Vert _{p'} \Vert \nabla E(f)\Vert _p \le 12\Vert x-\tfrac{1}{2}\Vert _p \Vert x-\tfrac{1}{2}\Vert _{p'} \Vert f\Vert _p. \end{aligned}$$

Here, we used our previous result in equation (4.5). If $p=\infty$ then

$$\begin{aligned} \Vert P\big (\nabla E(f)\big )\Vert _\infty =\sup _{x\in [0,1]} 12|x-\tfrac{1}{2}|\left| \int _0^1 (y-\tfrac{1}{2})\left( \int _0^y f(x)\,dx-\int _y^1 f(x)\,dx\right) dy\right| \\ =12\Vert x-\tfrac{1}{2}\Vert _\infty \left| \int _0^1 (y-\tfrac{1}{2})\left( \int _0^y f(x)\,dx-\int _x^1 f(x)\,dx\right) dy\right| \\ \le 12\Vert x-\tfrac{1}{2}\Vert _\infty \Vert x-\tfrac{1}{2}\Vert _1\left\| \int _0^x f(y)\,dy-\int _x^1 f(y)\,dy\right\| _\infty \\ \le 12\Vert x-\tfrac{1}{2}\Vert _\infty \Vert x-\tfrac{1}{2}\Vert _1\Vert f\Vert _\infty . \end{aligned}$$

Collecting the above results, we conclude that

$$\begin{aligned} \Vert (1-P)\nabla E(f)\Vert _p \le \Vert \nabla E(f)\Vert _p+\Vert P(\nabla E(f))\Vert _p \le \big (1+12\Vert x-\tfrac{1}{2}\Vert _p \Vert x-\tfrac{1}{2}\Vert _{p'}\big )\Vert f\Vert _p. \end{aligned}$$

This proves that $\Vert A(f)\Vert _p \le L\Vert f\Vert _p$ where $L=\big (1+12\Vert x-\tfrac{1}{2}\Vert _p \Vert x-\tfrac{1}{2}\Vert _{p'}\big ).$

For the regularity results, equation (2.8) immediately gives

4.6 $$\begin{aligned} \frac{d}{dx}\, \nabla E(f)(x) = 2f(x). \end{aligned}$$

This immediately implies (4.3) for the unconstrained selection gradient. Equation (4.6) also shows that if $f\in C^k[0,1]$ then $\nabla E(f)\in C^{k+1}[0,1]$, which gives (4.4) for the unconstrained selection gradient. For the constrained selection gradient we compute

$$\begin{aligned} \frac{d}{dx} A f(x) = 2 f(x) - 12 H(w(f)) \int _0 ^1 \left( y - \frac{1}{2} \right) \left( \int _0 ^y f(z) dz - \int _y ^1 f(z) dz\right) dy. \end{aligned}$$

This implies the mapping properties (4.3) and (4.4) for the constrained selection gradient as well. $\square$

To investigate further properties of the constrained selection gradient, we begin by computing that

$$\begin{aligned} \sup _{x\in [0,1]} \int _0^1 |k(x,y)|\,dy \not < 1. \end{aligned}$$

In the sense of Kress^33^, *A* is not a contraction. Therefore, existence and uniqueness of a solution to a Fredholm type integral equation cannot be established by Neumann series, since that would require that *A* is a contraction. It is however a compact mapping from $L^p[0,1]$ to $L^q[0,1]$ for all $p\in (1,\infty ]$ and $q\in [1,\infty )$.

#### Proposition 4.2

Both the constrained and unconstrained selection gradients are compact mappings from $L^p[0,1]$ to $L^q[0,1]$ for all $p>1,$ including $p=\infty$, and *q* such that $1\le q< \infty$.

#### Proof

Let $(X,\mu )$ be a positive measure space, and let $k:X\times X\rightarrow \mathbb {R}$ be a measurable function. For $p>1$ and $q<\infty$, define $p'=p/(1-p)$ and the “double norm” of *k* by

$$\begin{aligned} \Vert k\Vert = \left( \int _X\left( \int _X|k(r,s)|^{p'}\,d\mu (s)\right) ^{q/p'}d\mu (r)\right) ^{1/q} \end{aligned}$$

or if $p=\infty$ and $q=1$, then $\Vert k\Vert =\sup \{|k(x,y)|,\ x,y\in X\}$. If the double norm of *k* is finite, then it defines a compact kernel operator $L^p(X)\rightarrow L^q(X)$, see Jörgens^34^, page 275–277. The double norms of the kernels of both the unconstrained and the constrained selection gradients are finite. Here the space $X =[0,1]$ is the unit interval, and $\mu$ is the Lebesgue measure. $\square$

### Proofs for the function-valued adaptive dynamics

The following lemma shows that the monotonicity of a function has implications for its MCA. It will be useful in the proof of Theorem 2.4.

#### Lemma 4.3

Assume that *f* is a measurable, bounded, nonnegative function defined on the unit interval [0, 1], and assume that $0<\int _0^1 f(x)\,dx$. If *f* is increasing (decreasing) then its MCA is bigger (smaller) than or equal to $\frac{1}{2}$. If *f* is continuous and strictly increasing then its MCA is strictly bigger than $\frac{1}{2}$.

#### Proof

By the definition of MCA (2.4),

$$\begin{aligned} {{\,\textrm{MCA}\,}}(f)<\frac{1}{2} \iff \int _0^1 (x-\frac{1}{2}) f(x)\,dx < 0. \end{aligned}$$

By a change of variables, this is equivalent to

$$\begin{aligned} \int _{-1/2}^{0} f\left( t+\tfrac{1}{2}\right) t \,dt + \int _{0}^{1/2} f\left( t+\tfrac{1}{2}\right) t \,dt < 0. \end{aligned}$$

By another change of variables, this condition is equivalent to

$$\begin{aligned} \int _{0}^{1/2} \left( f\left( \tfrac{1}{2}+t\right) - f\left( \tfrac{1}{2}-t\right) \right) t \,dt < 0. \end{aligned}$$

If *f* is decreasing then $f(\tfrac{1}{2}+t)- f(\tfrac{1}{2}-t)\le 0$, and if *f* is increasing then $f(\tfrac{1}{2}+t)- f(\tfrac{1}{2}-t)\ge 0.$ If *f* is continuous and strictly increasing, then $f(\tfrac{1}{2}+t)- f(\tfrac{1}{2}-t)>0$ on a set of positive measure in [0, 1/2]. The conclusion follows. $\square$

As an application of the results on the unconstrained and constrained selection gradients and Lemma 4.3 we can now prove Theorem 2.4.

#### Proof of Theorem 2.4

We first assume that $w(f)\ge 0$. Then $H(w(f))=1$ in the definition (2.13). If $\lambda =0$, then we solve $Af=0$, which using (4.1) is equivalent to

$$\begin{aligned} 2\int _0^x f(y)\,dy - \int _0^1 f(y)\,dy = 12(x-\tfrac{1}{2}) \int _0^1 (y-\tfrac{1}{2}) \left( 2\int _0^y f(z)dz - \int _0 ^1 f(z) dz \right) \,dy. \end{aligned}$$

The right side is a differentiable function of *x*, so the left side is also, and differentiating both sides we obtain that *f*(*x*) is constant. This completes the proof in this case.

Assume that $\lambda \ne 0$. Taking the derivative of $\lambda f(x)=Af(x)$ we get

$$\begin{aligned} \lambda f'(x) = 2f(x)-12\int _0^1 \left( y-\frac{1}{2}\right) \left( \int _0^y f - \int _y^1 f\right) dy \end{aligned}$$

This is an integro-differential equation of Fredholm type with separable kernel, so the solution is found by setting the integral to some fixed real number $\beta$ and solve for $\beta$ at a later step, see Kress^33^. We obtain $\lambda f'-2f=\beta$. The solution is

$$\begin{aligned} f(x) = a e^{2x/\lambda } - \frac{\beta }{2} \end{aligned}$$

for some constant *a*. Inserting this into $\lambda f=Af$, we find

$$\begin{aligned} \lambda a e^{2x/\lambda } - \lambda \frac{\beta }{2} = a \frac{\lambda }{2}\left( 2e^{2x/\lambda }-1-e^{2/\lambda }\right) -12\left( x-\frac{1}{2}\right) \int _0^1 \left( y-\frac{1}{2}\right) \left( a\frac{\lambda }{2}\left( 2e^{2y/\lambda }-1-e^{2/\lambda }\right) \right) dy, \end{aligned}$$

so canceling $\lambda$ and subtracting $ae^{2x/\lambda }$ on both sides,

$$\begin{aligned} - \frac{\beta }{2} = a\left( -1-e^{2/\lambda }\right) -12a\left( x-\frac{1}{2}\right) \int _0^1 \left( y-\frac{1}{2}\right) \left( e^{2y/\lambda }-\frac{1}{2}-\frac{1}{2}e^{2/\lambda }\right) dy. \end{aligned}$$

Since $-1-e^{2/\lambda }$ is constant,

$$\begin{aligned} \int _0^1 \left( y-\frac{1}{2}\right) \left( -\frac{1}{2}-\frac{1}{2}e^{2/\lambda }\right) dy = 0. \end{aligned}$$

Furthermore, since $e^{2y/\lambda }$ is either increasing or decreasing (but never constant), we may apply Lemma 4.3 and obtain

$$\begin{aligned} \int _0^1 \left( y-\frac{1}{2}\right) e^{2y/\lambda }dy = K \ne 0, \end{aligned}$$

where $K=\frac{\lambda }{2}\left( \frac{1}{2}-\frac{\lambda }{2}\right) e^{2/\lambda }+\frac{\lambda }{2}\left( \frac{1}{2}+\frac{\lambda }{2}\right)$ is a constant. Thus,

$$\begin{aligned} - \frac{\beta }{2} = a\left( -1-2e^{2/\lambda }\right) -12aK\left( x-\tfrac{1}{2}\right) . \end{aligned}$$

The left hand side is a constant but the right hand side varies linearly with *x*. Therefore, it must be that $a=0$ and $\beta =0$. In the case ${{\,\textrm{MCA}\,}}(f)<\frac{1}{2}$, an almost identical computation proves that the only solution to $Af=\lambda f$ is $f=0$. $\square$

#### Proof of Lemma 2.6

Let $\alpha$ be a solution to (2.16) with *A* defined by (2.13) and denote by $\alpha _t$ the solution after time *t*. First, note that since $\alpha _t$ is a solution it is measurable, and $\alpha$ is a piecewise $C^1$-curve on $L^p[0,1]$, where *p* is the same as for the initial data. Consider the case $w(\alpha _t)<0$. Then $A=\nabla E$, which satisfies a Lipschitz condition on the $L^p[0,1]$ space. Thus^35^, we may compute

$$\begin{aligned} \frac{d}{dt} \int _0^1 \alpha _t(x)dx = \int _0^1 (A\alpha _t)(x)\,dx. \end{aligned}$$

Using $$A=\nabla E$$,

$$\begin{aligned} \int _0^1 (A\alpha _t)(x)\,dx = \int _0^1 \left( \int _0^x\alpha _t(y)dy - \int _x^1\alpha _t(y)dy\right) \,dx. \end{aligned}$$

The right hand side of this is simplified by (2.17) such that

$$\begin{aligned} \frac{d}{dt} \int _0^1 \alpha _t(x)dx = 2\left( \frac{1}{2}-{{\,\textrm{MCA}\,}}(\alpha _t)\right) \int _0^1\alpha _t(x)\,dx, \end{aligned}$$

so the denominator in the definition of the MCA, see (2.4), does not approach zero. Moreover,

$$\begin{aligned} \frac{\partial }{\partial t} \int _0^1 x\alpha _t(x)\,dx&= \int _0^1 x\left( \int _0^x\alpha _t(y)dy - \int _x^1\alpha _t(y)dy\right) dx\\&= \int _0^1 \left( x-\frac{1}{2}\right) \left( \int _0^x\alpha _t(y)dy - \int _x^1\alpha _t(y)dy\right) dx +\frac{1}{2}\int _0^1 \left( \int _0^x\alpha _t(y)dy- \int _x^1\alpha _t(y)dy\right) dx. \end{aligned}$$

The first term in this sum is simplified using integration by parts:

$$\begin{aligned} \int _0^1 \left( x-\frac{1}{2}\right) \left( \int _0^x\alpha _t(y)dy - \int _x^1\alpha _t(y)dy\right) dx = -\int _0^1 (x^2-x)\alpha _t(x)\, dx. \end{aligned}$$

If $\alpha _t$ is a strategy of the function-valued game, then according to the assumptions (2.4)

$$\begin{aligned} -\int _0^1 (x^2-x)\alpha _t(x)\, dx = \int _0^1 (x-x^2)\alpha _t(x)\, dx \ge 0. \end{aligned}$$

Collecting these results we obtain

$$\begin{aligned} \frac{d}{dt}{{\,\textrm{MCA}\,}}(\alpha _t)&= \left( \frac{d}{dt}\int _0^1 x\alpha _t(x)dx\right) \frac{1}{\int _0^1 \alpha _t(x)dx} - \left( \int _0^1 x\alpha _t(x)dx\right) \frac{1}{\left( \int _0^1 \alpha _t(x)dx\right) ^2}\left( \frac{d}{dt}\int _0^1 \alpha _t(x)dx\right) \\&=\left( \int _0^1 (x-x^2)\alpha _t(x)dx +\left( \frac{1}{2}-{{\,\textrm{MCA}\,}}(\alpha _t)\right) \int _0^1\alpha _t(x)dx \right) \frac{1}{\int _0^1 \alpha _t(x)dx} \\&- \left( \int _0^1 x\alpha _t(x)dx\right) \frac{1}{\left( \int _0^1 \alpha _t(x)dx\right) ^2}2\left( \frac{1}{2}-{{\,\textrm{MCA}\,}}(\alpha _t)\right) \end{aligned}$$

$$\begin{aligned}&=\frac{1}{\int _0^1 \alpha _t(x)\,dx}\int _0^1 (x-x^2)\alpha _t(x)dx +\left( \frac{1}{2}-{{\,\textrm{MCA}\,}}(\alpha _t)\right) -2{{\,\textrm{MCA}\,}}(\alpha _t)\left( \frac{1}{2}-{{\,\textrm{MCA}\,}}(\alpha _t)\right) \\&=2\left( \frac{1}{2}-{{\,\textrm{MCA}\,}}(\alpha _t)\right) ^2+\frac{1}{\int _0^1 \alpha _t(x)\,dx}\int _0^1 (x-x^2)\alpha _t(x)dx. \end{aligned}$$

This shows that the MCA of a strategy *f* such that $0<{{\,\textrm{MCA}\,}}(f)<\frac{1}{2}$ has a positive derivative with respect to time in the adaptive dynamics system.

Next, consider the case $w(\alpha _t)\ge 0$. Define $A_c$ by

$$\begin{aligned} A_cf(x)=\int _0^x f(y)\,dy- \int _x^1 f(y)\,dy -12(x-\tfrac{1}{2})\int _0^1 (y-\tfrac{1}{2})\left( \int _0^y f - \int _y^1 f\right) dy. \end{aligned}$$

Since $w(\alpha _t)\ge 0$, the operators *A* and $A_c$ coincide. Notice that $A_c$ is linear, and it is bounded on $L^p[0,1]$ by Lemma 4.1. Again we may differentiate under the integral with respect to *t*. Since $\int _0^1(x-\frac{1}{2})dx = 0$,

4.7 $$\begin{aligned} \frac{d}{dt} \int _0^1 \alpha _t(x)dx = \int _0^1 \left( \int _0^x\alpha _t(y)dy - \int _x^1\alpha _t(y)dy\right) \,dx. \end{aligned}$$

As in the previous case, the denominator of the MCA does not approach zero whenever $\alpha _t$ is a strategy. Moreover, by the definition of $A_c$,

$$\begin{aligned} \frac{d}{dt} \int _0^1 x\alpha _t(x)\,dx&= \\&\int _0^1 x\left( \int _0^x\alpha _t(y)dy - \int _x^1\alpha _t(y)dy\right. \left. -12 (x-\tfrac{1}{2})\int _0^1(y-\tfrac{1}{2})\left( \int _0^y \alpha _t(z)dz - \int _y^1 \alpha _t(z)dz\right) dy\right) dx. \end{aligned}$$

Let $q_t=\int _0^1(y-\tfrac{1}{2})\left( \int _0^y \alpha _t(z)dz - \int _y^1 \alpha _t(z)dz\right) dy$ and compute

$$\begin{aligned}&\int _0^1 x\left( \int _0^x\alpha _t(y)dy - \int _x^1\alpha _t(y)dy-12q_t(x-\tfrac{1}{2})\right) dx\\&= \int _0^1 \left( x-\frac{1}{2}\right) \left( \int _0^x\alpha _t(y)dy - \int _x^1\alpha _t(y)dy-12q_t(x-\tfrac{1}{2})\right) dx \\&+\frac{1}{2} \int _0^1 \left( \int _0^x\alpha _t(y)dy - \int _x^1\alpha _t(y)dy-12q_t(x-\tfrac{1}{2})\right) dx. \end{aligned}$$

Thus,

4.8 $$\begin{aligned} \frac{d}{dt} \int _0^1 x\alpha _t(x)\,dx&= \int _0^1 \left( x-\frac{1}{2}\right) \left( \int _0^x\alpha _t(y)dy - \int _x^1\alpha _t(y)dy\right) dx \nonumber \\&-12\underbrace{\int _0^1(x-\tfrac{1}{2})^2\,dx}_{=1/12}\int _0^1 \left( x-\frac{1}{2}\right) \left( \int _0^x\alpha _t(y)dy - \int _x^1\alpha _t(y)dy\right) dx \nonumber \\&+\frac{1}{2} \int _0^1 \left( \int _0^x\alpha _t(y)dy - \int _x^1\alpha _t(y)dy-12q_t(x-\tfrac{1}{2})\right) dx \nonumber \\&= \frac{1}{2} \int _0^1 \left( \int _0^x\alpha _t(y)dy - \int _x^1\alpha _t(y)dy\right) dx. \end{aligned}$$

The results in (4.7) and (4.8) together implies that

$$\begin{aligned} \frac{d}{dt} \int _0^1 \left( x-\tfrac{1}{2}\right) \alpha _t(x)\, dx = 0, \text { and thus } w(\alpha _t)\ge 0 \implies \frac{d}{dt} w(\alpha _t) = 0. \end{aligned}$$

The denominator of the MCA is not approaching zero, so this implies that the derivative of ${{\,\textrm{MCA}\,}}(\alpha _t)$ with respect to *t* is zero. Computing $\frac{d}{dt}{{\,\textrm{MCA}\,}}(\alpha _t)$ in the case $w(\alpha _t)\ge 0$ is just like in the case $w(\alpha _t)<0$ but with an additional term. The result for both cases together is

4.9 $$\begin{aligned} \frac{d}{dt}{{\,\textrm{MCA}\,}}(\alpha _t) = 2\left( \frac{1}{2}-{{\,\textrm{MCA}\,}}(\alpha _t)\right) ^2+\frac{1-H(w(\alpha _t))}{\int _0^1\alpha _t(x)dx}\int _0^1x(1-x)\alpha _t(x)dx. \end{aligned}$$

In particular,

$$\begin{aligned} {{\,\textrm{MCA}\,}}(\alpha _t)=1/2\ \implies \frac{d}{dt}{{\,\textrm{MCA}\,}}(\alpha _t) =0. \end{aligned}$$

If $\alpha _t$ is a strategy fulfilling (2.4), then the second term on the right side of (4.9) is non-negative, so if also $0<{{\,\textrm{MCA}\,}}(\alpha _t)<\frac{1}{2}$ then $\frac{d}{dt}{{\,\textrm{MCA}\,}}(\alpha _t)>0$. Removing the second part of the RHS in (4.9), a lower bound on ${{\,\textrm{MCA}\,}}(\alpha _t)$ is obtained. $\square$

Next we prove Theorem 2.7.

#### Proof of Theorem 2.7

The operator *A* (2.13) changes with *w* (2.9) which is used to express the MCA constraint. We analyze the situations $w(f_0)<0$ and $w(f_0)\ge 0$ separately. Starting with the latter, let us define $A_c:L^p[0,1]\rightarrow L^p[0,1]$ by

$$\begin{aligned} A_cf(x)=\int _0^x f(y)\,dy- \int _x^1 f(y)\,dy -12(x-\tfrac{1}{2})\int _0^1 (y-\tfrac{1}{2})\left( \int _0^y f - \int _y^1 f\right) dy. \end{aligned}$$

Notice that $A_c$ is linear on $L^p[0,1]$, and by applying Lemma 4.1 to $A_c$ we obtain a Lipschitz condition: $\Vert A_c(f)-A_c(g)\Vert \le L\Vert f-g\Vert$ for $L=(1+12\Vert x-\frac{1}{2}\Vert _p\Vert x-\frac{1}{2}\Vert _q)$ and for all $f,g\in L^p[0,1]$. Therefore, the Picard-Lindelöf theorem applies. We quote the theorem as stated by Brezis^36^:

**Theorem 7.3 of **^36^. Let *E* be a Banach space with norm $\Vert \cdot \Vert$ and $F:E\rightarrow E$ a Lipschitz mapping, i.e., there is a constant *L* such that $\Vert Fu-Fv\Vert \le L\Vert u-v\Vert$ for all $u,v\in E$. Given $u_0\in E$, there is a unique $C^1$-curve

$$\begin{aligned}u:[0,\infty )\rightarrow E\end{aligned}$$

satisfying the initial value problem

$$\begin{aligned} du/dt = F(u),\quad u(0)=u_0. \end{aligned}$$

Here, the mapping *F* corresponds to $A_c$, $L=(1+12\Vert x-\frac{1}{2}\Vert _p\Vert x-\frac{1}{2}\Vert _q)$ and $E=L^p[0,1]$. Since the Lipschitz condition on $A_c$ is independent of $f_0$ and global on $L^p[0,1]$, the solution $\alpha$ to the initial value problem ${\dot{\alpha }}=A_c\alpha ,\ \alpha (0)=f_0$ is defined on $t>0$ for any given initial data $f_0\in L^p[0,1]$.

Next, if $w(f_0)<0$ then $A=\nabla E$, so we would like to analyze the mapping $\nabla E :L^p[0,1]\rightarrow L^p[0,1]$ given by (2.8). Since $\nabla E$ is linear on $L^p[0,1]$ and bounded (by Lemma 4.1) we obtain a Lipschitz condition: $\Vert \nabla E(f)-\nabla E(g)\Vert _p \le \Vert f-g\Vert _p.$ Again, the Picard-Lindelöf theorem applies and we obtain existence and uniqueness of solutions, but this time the solution satisfies ${\dot{\alpha }}=\nabla E(\alpha ),\ \alpha (0)=f_0$.

Following the same computations as in the proof of Lemma 2.6,

$$\begin{aligned} w(\alpha _t)\ge 0\implies \frac{\partial }{\partial t} w(\alpha _t)= \frac{\partial }{\partial t} \int _0^1 (x-\tfrac{1}{2}) \alpha _t(x)dx = 0. \end{aligned}$$

That is, if the solution is such that $w(\alpha _t)\ge 0$ at some *t* then it continues to be such that $w(\alpha _t)\ge 0$. If on the other hand $w(\alpha _t)<0$ then it might happen that $w(\alpha _{t_1})=0$ at some later time $t_1$. Then we may solve ${\dot{\alpha }}=A\alpha$ on $t>t_1$ with $\alpha _{t_1}$ as initial condition to obtain a unique solution at later times. In conclusion, the initial value problem (2.16) admits a solution $\alpha :[0,\infty )\rightarrow L^p[0,1]$ for any initial data $f_0\in L^p[0,1]$.

As in the beginning of the proof of Lemma 2.6, we can conclude that if $\int _0^1 f_0(x)\,dx\ne 0$ for any initial data $f_0\in L^p[0,1]$ then $\int _0^1 \alpha _t(x)\,dx\ne 0$ for all *t*. Assuming that the initial data $f_0$ satisfies ${{\,\textrm{MCA}\,}}(f_0)=1/2$, Lemma 2.6 implies that the MCA of the solution curve $\alpha (t)$ is constant for all $t>0.$ Thus also the initial value problem with *A* instead of $A_c$, that is, ${\dot{\alpha }}=A\alpha ,\ \alpha (0)=f_0$ with ${{\,\textrm{MCA}\,}}(f_0)=\frac{1}{2}$ admits a solution with constant MCA.

Next, we prove that if $f_0$ is $C^k$-smooth then the solution $\alpha (t)$ is also $C^k$-smooth with respect to *x*. We first compute that the derivative of $A\alpha _t(x)$ with respect to *x* is

$$\begin{aligned} \frac{\partial }{\partial x} A\alpha _t(x) = 2\alpha _t(x) -12 H(w(\alpha _t))\int _0^1 \left( y-\frac{1}{2}\right) \left( \int _0^y \alpha _t(z)\,dz-\int _y^1 \alpha _t(z)\,dz\right) dy. \end{aligned}$$

If $\alpha _t$ is $C^k$-smooth with respect to *x*, then $A\alpha _t$ is $C^{k+1}$-smooth but then $\alpha _t$ is $C^{k+1}$-smooth. Notice that equation (2.16) can be cast into the integral form

4.10 $$\begin{aligned} \alpha (t) = f_0 + \int _0^t A\big (\alpha (s)\big )\, ds. \end{aligned}$$

This is no longer an integro-differential equation but rather an integral equation of mixed Volterra and Fredholm type. The existence of solutions to the initial value problem is transferred into a fixedpoint problem of (4.10). In this situation it is suitable to use the Banach fixed point theorem, or even better, the Picard-Lindelöf theorem. Using this integral form of the dynamics equation, it then follows that the initial data $f_0$ determines the smoothness of $\alpha (t)$ with respect to *x*.

Let $f_0\in L^p[0,1]$ with $p\ge 2$. To show that the solution’s $L^2$ norm is constant, let $p=2$ and compute the time derivative of the $L^2$ norm

$$\begin{aligned} \frac{d}{dt}\Vert \alpha (t)\Vert ^2 = \frac{d}{dt}\langle \alpha (t),\alpha (t)\rangle = 2\langle \dot{\alpha }(t),\alpha (t)\rangle =2\langle A\alpha (t),\alpha (t)\rangle . \end{aligned}$$

Let $$q_0=\int _0^1 (y-\frac{1}{2})(\int _0^y \alpha _t(x)\,dx-\int _y^1 \alpha _t(x)\,dx)dy$$. Then

$$\begin{aligned} \frac{d}{dt}\Vert \alpha (t)\Vert ^2&= 2\int _0^1 \alpha _t(x) \left( \int _0^x \alpha _t(x)\,dx -\int _x^1 \alpha _t(x)\,dx - 12q_0 H(w(f))(x-\tfrac{1}{2})\right) \,dx\\&=2\underbrace{E[\alpha (t),\alpha (t)]}_{=0}-24H(w(f))q_0\int _0^1(x-\tfrac{1}{2})\alpha _t(x)\,dx \end{aligned}$$

4.11 $$\begin{aligned} \implies \frac{d}{dt}\Vert \alpha (t)\Vert = \frac{-12q_0 H(w(f))\int _0^1(x-\tfrac{1}{2})\alpha _t(x)\,dx}{\Vert \alpha (t)\Vert }. \end{aligned}$$

Here, we used that $E[f,f]=0$ for all $f\in L^1[0,1]\supset L^p[0,1]$. Notice that $q_0\ge 0$ by (4.2). Recall from the first part of the proof that if ${{\,\textrm{MCA}\,}}(f_0)=\frac{1}{2}$ then also ${{\,\textrm{MCA}\,}}(\alpha _t)=\frac{1}{2}.$ Since $\int _0^1(x-\tfrac{1}{2})\alpha _t(x)\,dx=0$ is equivalent to ${{\,\textrm{MCA}\,}}(\alpha _t)=\frac{1}{2}$, a consequence of (4.11) is

$$\begin{aligned} \frac{d}{dt}\Vert \alpha (t)\Vert = 0. \end{aligned}$$

That is, the solution $\alpha (t)$ has constant $L^2$ norm. $\square$

We now prove Lemma 2.8 which shows that the adaptive dynamics evolves to defeat the initial data, at least for small time.

#### Proof of Lemma 2.8

First consider the case ${{\,\textrm{MCA}\,}}(f_0)<\frac{1}{2}$. Since $f_0$ is non-negative and not constant, $\int _0^1 f_0(x)\,dx>0$. The solution curve $\alpha$ is continuous, so for sufficiently small *t*, $\int _0^1 \alpha _t(x)\,dx>0$. The constraint ${{\,\textrm{MCA}\,}}(f_0)<\frac{1}{2}$ is equivalent to

$$\begin{aligned} \int _0^1 (x-\tfrac{1}{2}) f_0(x)\,dx<0, \end{aligned}$$

and again, for small *t*, this holds also for $\alpha _t$ by the continuity of the solution curve. That is, there exist a constant $b>0$ and an open interval $-b<t<b$ such that ${{\,\textrm{MCA}\,}}(\alpha _t)<\frac{1}{2}$ for all $t\in (-b,b)$. Then,

$$\begin{aligned} \frac{d}{dt} E[\alpha _t,f_0] \,&= \int _0^1 A\alpha _t (x)\left( \int _0^x f_0(y)\,dy - \int _x^1 f_0(y)\,dy\right) dx\\&= \int _0^1 \left( \int _0^x \alpha _t (y)\,dy-\int _x^1 \alpha _t (y)\,dy\right) \left( \int _0^x f_0(y)\,dy - \int _x^1 f_0(y)\,dy\right) dx\\&=\langle \nabla E(\alpha _t), \nabla E(f_0)\rangle . \end{aligned}$$

Thus,

4.12 $$\begin{aligned} \left. \frac{d}{dt} E[\alpha _t,f_0]\right| _{t=0} =\langle \nabla E(f_0), \nabla E(f_0)\rangle>0. \end{aligned}$$

Consider *t* in the interval $-b<t<b$ such that ${{\,\textrm{MCA}\,}}(\alpha _t)<\frac{1}{2}$. Then $A(\alpha _t)=\nabla E(\alpha _t)$. The function $t\mapsto \langle \nabla E(\alpha _t), \nabla E(f_0)\rangle$ is continuous, since the inner product $\langle \,,\rangle$ is continuous and $\nabla E$ is Lipschitz continuous. Since the derivative of $E[\alpha (t),f_0]$ is positive at $t=0$, by (4.12), and continuous, there exists a time $t>0$ such that $E[\alpha (t),f_0]>0.$

Second, consider the case ${{\,\textrm{MCA}\,}}(f_0)=\frac{1}{2}$. Then the selection gradient is $A(f_0)=(1-P)\nabla E(f_0)$. By construction, then, ${{\,\textrm{MCA}\,}}(\alpha _t)=\frac{1}{2}$ for all $t\ge 0$. Then,

$$\begin{aligned} \frac{d}{dt} E[\alpha _t,f_0]&= \int _0^1 A\alpha _t (x)\left( \int _0^x f_0(y)\,dy - \int _x^1 f_0(y)\,dy\right) dx\\&= \int _0^1 \left( \int _0^x \alpha _t (y)\,dy-\int _x^1 \alpha _t (y)\,dy\right) \left( \int _0^x f_0(y)\,dy - \int _x^1 f_0(y)\,dy\right) dx\\ \quad&-12\int _0^1(x-\tfrac{1}{2})\alpha _t(x) dx \int _0^1(x-\tfrac{1}{2}) \left( \int _0^x f_0(y)\,dy - \int _x^1 f_0(y)\,dy\right) dx\\&= \langle A(\alpha (t)),\nabla E(f_0)\rangle \end{aligned}$$

Since ${{\,\textrm{MCA}\,}}(\alpha _t)=\frac{1}{2}$ for all at all positive times, $\Vert A(\alpha _{t}-\alpha _{t'})\Vert < K\Vert \alpha _{t}-\alpha _{t'}\Vert$ for $t,t'>0$ for some $K>0$ by Lemma 4.1. It follows that $t\mapsto \langle A(\alpha (t)),\nabla E(f_0)\rangle$ is continuous. At $t=0$ we have $\alpha (0)=f_0$ and

$$\begin{aligned} E[A(f_0),f_0] = \langle A(f_0),\nabla E(f_0)\rangle =\frac{1}{\Vert \tfrac{1}{2}-x\Vert ^2}\left( \Vert \tfrac{1}{2}-x\Vert ^2\Vert \nabla E(f_0)\Vert ^2-\langle \tfrac{1}{2}-x,\nabla E(f_0)\rangle ^2 \right) \end{aligned}$$

By the Cauchy-Schwarz inequality,

4.13 $$\begin{aligned} \Vert \tfrac{1}{2}-x\Vert ^2\Vert \nabla E(f_0)\Vert ^2\ge \langle \tfrac{1}{2}-x,\nabla E(f_0)\rangle ^2 \end{aligned}$$

with equality if and only if $a(\frac{1}{2}-x)=b\nabla E(f_0)(x)$ for non-zero constants *a*, *b*. Hence by the Cauchy-Schwarz inequality, $E[A(f_0),f_0]$ is positive or zero, and it is zero if and only if $a(\frac{1}{2}-x)=b\nabla E(f_0)(x)$. Since $a(\frac{1}{2}-x)=b\nabla E(f)(x)$ only if *f* is constant almost everywhere, the inequality (4.13) is strict whenever $f_0$ is not constant. $\square$

### Proofs for the vector-valued adaptive dynamics

#### Proof of Lemma 2.12

In Gauss’s algorithm, we replace row *j* with row *j* minus row $j+1$ for all $1\le j\le M$ as shown below.

$$\begin{aligned} \left. \begin{matrix} 0 & -1 & -1 & ... & -1\\ 1 & 0 & -1 & ... & -1\\ 1 & 1 & 0 & ... & -1\\ \vdots & & & & \vdots \\ 1 & 1 & 1 & ... & 0 \end{matrix} \ \right| \begin{matrix} 0 \\ 0 \\ 0 \\ \vdots \\ 0 \end{matrix} \iff \left. \begin{matrix} -1 & -1 & 0 & 0 & ... & 0\\ 0 & -1 & -1 & 0 & ... & 0\\ 0 & 0 & -1 & -1& ... & 0\\ \vdots & & & & & \vdots \\ 1 & 1 & 1 & 1 & ... & 0 \end{matrix} \ \right| \begin{matrix} 0 \\ 0 \\ 0 \\ \vdots \\ 0 \end{matrix} \end{aligned}$$

If $M+1$ is even, we add row 1, 3, and all of the odd rows up to row *M* to the last row. If $M+1$ is odd, we add row 1, 3, and all of the odd rows up to row $M-1$ to the last row. In this way we obtain

$$\begin{aligned} \left. \begin{matrix} -1 & -1 & 0 & 0 & ... & 0 & 0\\ 0 & -1 & -1 & 0 & ... & 0 & 0\\ 0 & 0 & -1 & -1& ... & 0 & 0\\ \vdots & & & & & & \vdots \\ 0 & 0 & 0 & 0 & ... & -1 & -1\\ 0 & 0 & 0 & 0 & ... & 0 & a \end{matrix} \ \right| \begin{matrix} 0 \\ 0 \\ 0 \\ \vdots \\ 0 \\ 0 \end{matrix} \quad \text {where } a= {\left\{ \begin{array}{ll} -1 & \text {when } M+1\text { is even,}\\ 0 & \text {when } M+1\text { is odd.} \end{array}\right. } \end{aligned}$$

We can deduce from this calculation that the kernel of *L* when $M+1$ is odd is the span of the vector $(1,-1,1,-1,...,-1,1)$. $\square$

#### Proof of Proposition 2.13

Consider the rank of $(I-P)L$. If $M+1$ is even, then $L: \mathbb {R}^{M+1} \rightarrow \mathbb {R}^{M+1}$ is a surjection. The matrix $I-P$ projects onto the orthogonal complement of the vector $\varvec{w}$, an *M*-dimensional subspace, so in this case the rank of $(I-P)L$ is *M*. If $M+1$ is odd, then *L* maps $\mathbb {R}^{M+1}$ to an *M*-dimensional subspace. Each of the columns of *L* are contained in this subspace. Considering just the first column, it is not orthogonal to $\varvec{w}$, it follows that $L \mathbb {R}^{M+1}$ is not contained in the orthogonal complement of $\varvec{w}$. Consequently, when we apply $(I-P)$ to $L \mathbb {R}^{M+1}$ the resulting subspace loses one dimension and is thus of dimension $M-1$. The proof then follows from the rank-nullity theorem in both cases. $\square$

#### Proof of Proposition 2.14

Since *Q* is a projection matrix, there exists a change of basis, implemented by a unitary matrix *U* under which *Q* has the form

$$\begin{aligned} U^T Q U = \begin{bmatrix} I_{k} & \varvec{0} \\ \varvec{0} & \varvec{0} \end{bmatrix} \end{aligned}$$

where $I_k$ is a $k\times k$ identity matrix, with *k* equal to the rank of *Q*. Then using this change of basis, we have

$$\begin{aligned} U^T (QS) U = U^T Q U U^T S U = (U^T Q U)(U ^T S U) = \begin{bmatrix} I_{k} & \varvec{0} \\ \varvec{0} & \varvec{0} \end{bmatrix} (U^T S U) = \begin{bmatrix} S' & S'' \\ \varvec{0} & \varvec{0} \end{bmatrix}. \end{aligned}$$

where $S'$ is the upper-left $k\times k$ block of $U^T S U$ and $S''$ is the upper-right corner of $U^T S U$ of size $k\times (n-k)$. Notice that $S'$ is also anti-symmetric, because anti-symmetric matrices are anti-symmetric with respect to any basis. Now, if *Q* and *S* are $n \times n$, then we compuate that

$$\begin{aligned} \det \begin{bmatrix} \lambda I_k-S' & S'' \\ \varvec{0} & \lambda I_{n-k} \end{bmatrix} = \lambda ^{n-k}\det (\lambda I_k-S') \end{aligned}$$

Since $S'$ is anti-symmetric, this polynomial has roots in $i\mathbb {R}$, and these roots are eigenvalues of *QS*. The remaining eigenvalue is 0, if $n-k>0$. $\square$

#### Proof of Corollary 2.15

We note that $(I-P)L$ has all real entries, and so the characteristic polynomial $\det (\lambda I - (I-P)L)$ has real coefficients. It therefore follows that if *z* is a root of this polynomial, which is equivalent to being an eigenvalue of $(I-P)L$, then ${\overline{z}}$ is also a root of this polynomial. By the preceding proposition, the eigenvalues of $(I-P)L$ are contained in $i \mathbb {R}$. The non-zero ones therefore occur in pairs of the form $\pm i b$ for non-zero $b \in \mathbb {R}$. The dimension of the eigenspace of the eigenvalue zero follows from Proposition 2.13. In the same way we apply the Proposition to $L=IL$, with *I* the identity matrix of the same dimensions as *L*, noting that this is a projection matrix, so the proposition applies in the same way. $\square$

#### Proof of Proposition 2.16

Let $\varvec{v}$ be either of the solutions in the statement of the proposition. We compute

$$\begin{aligned} L \varvec{v}= -\frac{M}{2} \begin{bmatrix} 1 \\ 1 \\ \vdots \\ 1 \\ 1 \end{bmatrix} + \begin{bmatrix} 0 \\ 1 \\ 2 \\ \vdots \\ M \end{bmatrix}. \end{aligned}$$

Since the ${{\,\textrm{MCA}\,}}$ of the first vector is equal to 1/2, it is orthogonal to $\varvec{w}$, and so left multiplication with *P* yields the zero vector. Let $\varvec{u}$ be the second vector.

Then

$$\begin{aligned} P\varvec{u} = \begin{bmatrix} 0 \\ 1 \\ 2 \\ \vdots \\ M \end{bmatrix} - \frac{M}{2} \begin{bmatrix} 1 \\ 1 \\ \vdots \\ 1 \\ 1 \end{bmatrix} \end{aligned}$$

The conclusion is $(I-P)L\varvec{v}=L\varvec{v}- PL\varvec{v}=(0,0,...,0)$. Since these $\varvec{v}_o$ and $\varvec{v}_e$ are linearly independent and since $\dim \textrm{Ker}\, (I-P)L=2$ by Proposition 2.13, they constitute a basis. $\square$

#### Proof of Proposition 2.17

Let $\varvec{v}_2$ be as in the statement of the proposition and compute

$$\begin{aligned} L \varvec{v}_2 = -(M-1) \begin{bmatrix} 1 \\ 1 \\ \vdots \\ 1 \\ 1 \end{bmatrix} + 2 \begin{bmatrix} 0 \\ 1 \\ 2 \\ \vdots \\ M \end{bmatrix} = 2M \varvec{w}+ \varvec{v}_1. \end{aligned}$$

Then, since ${{\,\textrm{MCA}\,}}(\varvec{v}_1) = 1/2$ it is in the orthogonal complement of the span of $\varvec{w}$. Consequently, since $I-P$ projects onto the orthogonal complement of $\varvec{w}$, $(I-P)L\varvec{v}_2= \varvec{v}_1$. We then also compute that

$$\begin{aligned} L \varvec{v}_1 = -2M \varvec{w}\implies (I-P)L\varvec{v}_1 = 0.\end{aligned}$$

$$\square$$

#### Proof of Theorem 2.19

We compute the characteristic polynomial of *L* using induction on the size of *L*. Let *L* be as in (2.20) of size $(n+1)\times (n+1)$ for some positive integer *n* and let $L_n$ be the same matrix but of size $n\times n$. We begin by subtracting the $(j+1)^{st}$ row from the $j^{th}$ row starting from the first row and continuing to the last row, keeping the last row unchanged. Then we calculate the determinant by expanding along the first column obtaining

4.14 $$\begin{aligned}&\det (L-\lambda I)= \det \begin{bmatrix} -\lambda & -1 & -1 & ... & -1\\ 1 & -\lambda & -1 & ... & -1\\ 1 & 1 & -\lambda & ... & -1\\ \vdots & & & \ddots & \vdots \\ 1 & 1 & 1 & ... & -\lambda \end{bmatrix} \nonumber \\&= \det \begin{bmatrix} -1-\lambda & \lambda -1 & 0 & ... & 0\\ 0 & -1-\lambda & \lambda -1 & \ddots & \vdots \\ 0 & 0 & \ddots & \ddots & 0\\ \vdots & & & -1-\lambda & \lambda -1\\ 1 & 1 & \dots & 1 & -\lambda \end{bmatrix} \nonumber \\&=(-1-\lambda ) \det (L_{n}-\lambda I_{n}) + (-1)^{n+2}\det \begin{bmatrix} \lambda -1 & 0 & ... & 0\\ -1-\lambda & \lambda -1 & ... & 0\\ 0 & \ddots & \ddots & \vdots \\ \vdots & & -1-\lambda & \lambda -1 \end{bmatrix} \nonumber \\&=(-1-\lambda ) \det (L_{n}-\lambda I_{n}) + (-1)^{n+2}(-1+\lambda )^n. \end{aligned}$$

We claim that

4.15 $$\begin{aligned} \det (L-\lambda I) = \sum _{k=0} ^{\frac{n+1}{2}} {n+1 \atopwithdelims ()2k} \lambda ^{2k}, \text { if n+1 is even}, \end{aligned}$$

and

4.16 $$\begin{aligned} \det (L-\lambda I) = - \lambda \sum _{k=0} ^{ \frac{n}{2}} {n+1 \atopwithdelims ()2k+1} \lambda ^{2k}, \text { if n+1 is odd.} \end{aligned}$$

Once these expressions are established, it is immediately apparent that 0 is not an eigenvalue of $L-\lambda I$ when $n+1$ is even, and it is an eigenvalue of algebraic multiplicity one when $n+1$ is odd. It is also apparent that all other eigenvalues are purely imaginary and occur in conjugate pairs. So, to complete the proof, we demonstrate (4.15) and (4.16).

We calculate directly that

$$\begin{aligned} \det \begin{bmatrix} -\lambda & -1\\ 1 & -\lambda \end{bmatrix} =\lambda ^2 +1,\quad \det \begin{bmatrix} -\lambda & -1 & -1\\ 1 & -\lambda & -1\\ 1 & 1 & -\lambda \end{bmatrix} =-\lambda ^3 -3\lambda . \end{aligned}$$

This demonstrates the base cases. Using (4.14) and the induction assumption for *n* odd, we compute $\det (L-\lambda I)$ in dimension $n+1 \times n+1$ is

$$\begin{aligned}(1+\lambda ) \sum _{k=0} ^{\frac{n-1}{2}} {n \atopwithdelims ()2k+1} \lambda ^{2k+1} + (-1)^n \sum _{j=0} ^n {n \atopwithdelims ()j} (-1)^{n-j} \lambda ^j\\ = \sum _{k=0} ^{\frac{n-1}{2}} {n \atopwithdelims ()2k+1} \lambda ^{2k+1} + \sum _{k=0} ^{\frac{n-1}{2}} {n \atopwithdelims ()2k+1} \lambda ^{2k+2} + \sum _{j=0} ^n {n \atopwithdelims ()j} (-1)^{j} \lambda ^j.\end{aligned}$$

We split the sum

$$\begin{aligned}\sum _{j=0} ^n {n \atopwithdelims ()j} (-1)^{j} \lambda ^j = - \sum _{k=0} ^{\frac{n-1}{2}} {n \atopwithdelims ()2k+1} \lambda ^{2k+1} + \sum _{k=0} ^{\frac{n-1}{2}} {n \atopwithdelims ()2k} \lambda ^{2k}. \end{aligned}$$

This shows that the determinant simplifies to

$$\begin{aligned} \sum _{k=0} ^{\frac{n-1}{2}} {n \atopwithdelims ()2k+1} \lambda ^{2k+2} + \sum _{k=0} ^{\frac{n-1}{2}} {n \atopwithdelims ()2k} \lambda ^{2k}. \end{aligned}$$

We re-index the first sum by setting $j=k+1$ and obtain

$$\begin{aligned} \sum _{j=1} ^{\frac{n+1}{2}} {n \atopwithdelims ()2j-1} \lambda ^{2j} + \sum _{k=0} ^{\frac{n-1}{2}} {n \atopwithdelims ()2k} \lambda ^{2k} = 1 + \lambda ^{n+1} + \sum _{k=0} ^{\frac{n-1}{2}} \left[ {n \atopwithdelims ()2k} + {n\atopwithdelims ()2k-1} \right] \lambda ^{2k}\\ = 1 + \lambda ^{n+1} + \sum _{k=0} ^{\frac{n-1}{2}} {n+1 \atopwithdelims ()2k} \lambda ^{2k} = \sum _{k=0} ^{\frac{n+1}{2}} {n+1 \atopwithdelims ()2k} \lambda ^{2k}.\end{aligned}$$

Since *n* is odd, $n+1$ is even, and this is indeed (4.15).

Next, assume our claim holds for *n* even, and we calculate using (4.14) and the induction assumption:

$$\begin{aligned} \det (L-\lambda I) = -(1+\lambda ) \sum _{k=0} ^{\frac{n}{2}} {n \atopwithdelims ()2k} \lambda ^{2k} + (-1)^n \sum _{j=0} ^n {n \atopwithdelims ()j} (-1)^{n-j} \lambda ^j \\ = - \sum _{k=0} ^{\frac{n}{2}} {n \atopwithdelims ()2 k} \lambda ^{2k} - \lambda \sum _{k=0} ^{\frac{n}{2}} {n \atopwithdelims ()2 k} \lambda ^{2k} + \sum _{j=0} ^n {n \atopwithdelims ()j} (-1)^j \lambda ^j. \end{aligned}$$

Splitting the sum in *j*, the first summand cancels resulting in the simplification to

$$\begin{aligned} - \lambda \sum _{k=0} ^{\frac{n}{2}} {n \atopwithdelims ()2 k} \lambda ^{2k} - \sum _{j=0} ^{n/2-1} {n \atopwithdelims ()2j+1} \lambda ^{2j+1} \\ = - \lambda - \lambda ^{n+1} - \lambda \sum _{k=1} ^{n/2-1} \left[ {n \atopwithdelims ()2k} + {n \atopwithdelims ()2k+1} \right] \lambda ^{2k} \\ = - \lambda - \lambda ^{n+1} - \lambda \sum _{k=1} ^{n/2-1} {n+1 \atopwithdelims ()2k+1} \lambda ^{2k} = - \lambda \sum _{k=0} ^{n/2} {n+1 \atopwithdelims ()2k+1} \lambda ^{2k}. \end{aligned}$$

This is indeed (4.16). $\square$

#### Proof of Proposition 2.20

Notice that

4.17 $$\begin{aligned} {{\,\textrm{MCA}\,}}(\varvec{y}) < \frac{1}{2} \iff 0> \sum _{j=0}^M (2j-M) y_{j} = -\sum _{j=0}^{M} (L\varvec{y})_j = - \frac{d}{dt} \sum _{j=0} ^M y_j. \end{aligned}$$

Therefore, it follows from ${{\,\textrm{MCA}\,}}(\varvec{y})<\frac{1}{2}$ that $\sum y_j$ is increasing. Next, we will show that the MCA increases as *t* increases.

Since $${{\,\textrm{MCA}\,}}(\varvec{y})<\frac{1}{2}$$,

$$\begin{aligned} \sum _{j=0}^M (j/M) y_j <\frac{1}{2}\sum _{j=0}^M y_j. \end{aligned}$$

Then using this equation together with ${\dot{\varvec{y}}} = L \varvec{y}$ and the definition of the MCA, we compute

$$\begin{aligned} \frac{d}{dt}{{\,\textrm{MCA}\,}}(\varvec{y}(t))&= \frac{\left( \sum _i (i/M) (L\varvec{y})_i\right) \left( \sum _j y_j\right) -\left( \sum _i (i/M)y_i\right) \left( \sum _j (L\varvec{y})_j\right) }{\left( \sum _k y_k\right) ^2}\\&>\frac{\sum _i (i/M-\frac{1}{2})(L\varvec{y})_i}{\sum _k y_k} \end{aligned}$$

It remains to prove that $\sum _i (i/M-\frac{1}{2})(L\varvec{y})_i\ge 0$. By the definition of *L*,

$$\begin{aligned} \sum _i (i/M)(L\varvec{y})_i =&\phantom {+}\,\frac{0}{M}(-y_1-y_2-...-y_M)\\&+\frac{1}{M}(y_0-y_2-...-y_M)\\&+\frac{2}{M}(y_0+y_1-y_3-...-y_M)\\&+...\\&+\frac{M}{M}(y_0+y_2+...+y_{M-1})\\ =&\sum _{i=0}^M y_i \left( \frac{M(M+1)}{2M}-\frac{(i+1)i}{2M}-\frac{i(i-1)}{2M}\right) \\ =&\sum _{i=0}^M y_i \left( \frac{M(M+1)}{2M}-\frac{2i^2}{2M}\right) . \end{aligned}$$

Using equation (4.17),

$$\begin{aligned} \sum _i \left( (i/M)-\frac{1}{2}\right) (L\varvec{y})_i =&\sum _{i=0}^M y_i\left( \frac{M(M+1)}{2M}-\frac{2i^2}{2M}-\frac{M}{2}+i\right) \\ =&\sum _{i=0}^M y_i\left( \frac{1}{2}-\frac{i^2}{M}+i\right) \\ =&\sum _{i=0}^M y_i\left( \frac{1}{2}+\frac{(M-i)i}{M}\right) . \end{aligned}$$

We conclude that

$$\begin{aligned} \frac{d}{dt}{{\,\textrm{MCA}\,}}(\varvec{y}(t))> \frac{1}{\sum _k y_k}\left( \sum _{i=0}^M y_i\left( \frac{1}{2}+\frac{(M-i)i}{M}\right) \right)>0. \end{aligned}$$

The last inequality follows from the assumption that $y_k \ge 0$ for all *k* and $\sum _k y_k> 0$. $\square$

### Python implementation

We provide a Python module that can be used for solving the adaptive dynamics problem numerically. The datasets generated and/or analysed during the current study are available in the GitHub repository,https://github.com/carljoar/adaptivegame

## Data Availability

We provide a Python module that can be used for solving the adaptive dynamics problem numerically. The datasets generated and/or analysed during the current study are available in the GitHub repository, (https://github.com/carljoar/adaptivegame)
